# 10-Year Trends in Heart Failure Among Patients With Dialysis and Nondialysis CKD

**DOI:** 10.1016/j.ekir.2026.106695

**Published:** 2026-07-04

**Authors:** Roosa Lankinen, Shunsuke Murata, Juan-Jesús Carrero, Karin Modig, Marie Evans, Anne-Laure Faucon

**Affiliations:** 1 Department of Medical Epidemiology and Biostatistics, Karolinska Institutet, Stockholm, Sweden; 2 Unit of Epidemiology, Institute of Environmental Medicine, Karolinska Institutet, Stockholm, Sweden; 3 Frontier Research Institute for Interdisciplinary Sciences, Tohoku University, Sendai, Japan; 4 Department of International and Community Oral Health, Tohoku University Graduate School of Dentistry, Sendai, Japan; 5 Department of Clinical Sciences, Danderyd Hospital, Stockholm, Sweden; 6 Department of Nephrology, Karolinska University Hospital, Stockholm, Sweden; 7 Department of Clinical Science, Intervention and Technology, Karolinska Institutet, Stockholm, Sweden

**Keywords:** CKD, diuretics, dialysis, epidemiology, heart failure, temporal trends

## Abstract

**Introduction:**

This study explores the rates and temporal trends of heart failure (HF) in advanced chronic kidney disease (CKD) compared with the general population (GP).

**Methods:**

We included all patients with nondialysis dependent CKD stage G4 (*n* = 25,591) and G5 (*n* = 13,968), on hemodialysis (HD; *n* = 10,635) and peritoneal dialysis (PD; *n* = 4511) enrolled in the Swedish Renal Registry during 2011 and 2021. Outcomes included HF-related hospitalization and HF-related mortality. Patients were followed until the event, death, or progression to a more severe CKD-stage and/or change of dialysis modality. Poisson models were used to estimate unadjusted incidence rates (IR) and IR standardized to an age- sex-matched GP.

**Results:**

Rates of HF-related hospitalization increased with more advanced stages of CKD, ranging from 3.7 to 6.8 times higher than those in the GP. Between 2011 and 2021, renin-angiotensin system inhibitor (RASi) and beta-blocker use remained stable and relatively low (44.5% to 70% for RASi), whereas diuretic prescription declined in nondialysis (ND)-CKD. This paralleled an important increase in the rates of HF-related hospitalization in CKD stage 4 (+51%), 5-ND (+36%), and HD (+30%), with a larger increase than that observed in the GP. In contrast, HF-related mortality steadily declined in both ND-CKD (up to -20.8% in CKD stage 4) and dialysis dependent CKD (up to -66% in HD), although slower than in the GP.

**Conclusion:**

Despite decline in HF-related mortality over the past decade—although slower than in the GP—HF-related hospitalization rates have risen substantially in advanced CKD, with a faster increase than those in the GP. These findings highlight the growing HF burden in CKD, and the need for targeted prevention and management strategies.

## Graphical abstract

**Figure:**
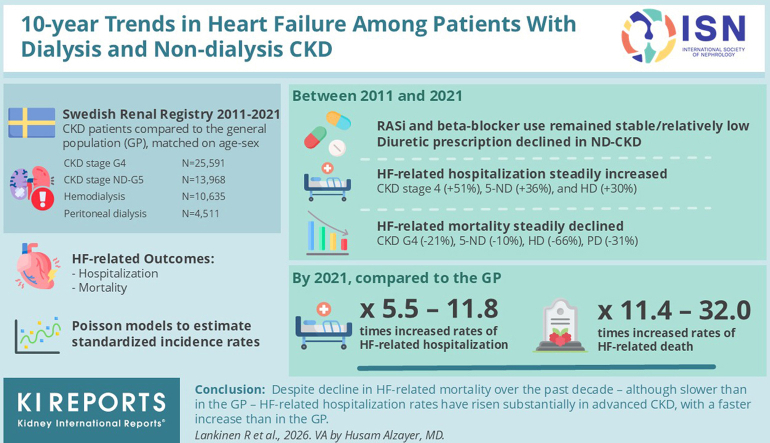

Cardiovascular disease (CVD) is the leading cause of mortality in patients with CKD, accounting for approximately 40% to 50% of all deaths in this population.^1^ Among CVD subtypes, HF is the most frequent cardiovascular complications in patients with CKD, with a prevalence increasing with worsening kidney function, ranging from 21.5% in stages 1 and 2, 41.3% in stages 4 and 5, and up to 44% in dialysis.^2^^,^^3^ Conversely, more than 50% of patients with HF have coexisting CKD.^4^^,^^5^ The co-occurrence of HF and CKD often leads to accelerated deterioration of both cardiac and renal functions,^6^, ^7^, ^8^ resulting in a markedly elevated risk of morbidity and mortality.^9^, ^10^, ^11^, ^12^

The interplay between the heart and the kidney is inherently complex, with dysfunction in 1 organ often adversely affecting the other.^9^^,^^13^^,^^14^ This makes the clinical management of these patients particularly challenging, especially because most of the patients with advanced CKD have been excluded from the pivotal trials evaluating HF management.^15^ As a result, guideline-directed HF therapies were extrapolated from studies conducted in patients without CKD or with earlier stages of CKD.^16^ The use of guideline-directed HF therapies in advanced CKD remains suboptimal, as concerns^17^ about hyperkalemia, worsening kidney function, and hypotension often lead to under-dosing or discontinuation, despite their well-established cardiorenal protective effects.^18^^,^^19^ Moreover, diuretics remain the cornerstone of volume management,^20^^,^^21^ yet their use in CKD is often inconsistent and/or underprescribed, partly because of concerns about their potential detrimental effects on CKD progression.^22^

Although several studies have documented HF trends in the general population^23^, ^24^, ^25^, ^26^, ^27^ or in patients with HF,^4^^,^^5^^,^^28^ data specific to patients with advanced CKD remain scarce,^29^^,^^30^ and often lacking standardization with the GP.^30^ Previous epidemiological studies conducted in CKD have predominantly focused on the bidirectional relationship between CKD and HF,^12^^,^^13^^,^^31^^,^^32^ without exploring temporal trends. The few studies that have examined temporal trends in HF in CKD were 2 decades old and only conducted in patients with kidney failure.^29^^,^^30^ To our knowledge, no study has comprehensively evaluated trends in HF in patients with ND-CKD, and comparative analyses between dialysis modalities are also lacking.

Using the nationwide Swedish Renal Registry (SRR), we explored rates and temporal trends of HF-related hospitalizations and HF-related mortality among patients with advanced CKD under nephrology care. In addition, to provide a more comprehensive picture of the evolving burden and characteristics of HF in advanced CKD, we also analyzed prescription patterns of key HF therapies, and explored the underlying causes of HF-related mortality.

## Methods

### Population Sources

The SRR is a nationwide quality registry of patients attending nephrology specialist care in Sweden.^33^ Nearly all nephrology clinics in Sweden (98%) report to the SRR with a national coverage of 75% to 96% of all eligible patients with CKD stage 4 and 5 and near to 100% of patients on maintenance dialysis. The SRR collects longitudinal information on a large set of clinical and biological data, as well as information on transition from ND-CKD to kidney replacement therapy or changing kidney replacement therapy modality (i.e., between dialysis modalities, from dialysis to kidney transplantation, and back to dialysis after kidney transplantation failure).

Through each citizen’s unique personal identification number, the SRR is linked to other government-run registries including the following: the *National Patient Registry* which provides information on all out-patient specialist consultations and hospitalizations occurring in Swedish healthcare since 1997; the *Prescribed Drug Registry*^34^ which records information on prescribed drugs dispensed at any Swedish pharmacy; and the *Death Registry*^35^ which provides information on date and causes of death. Government-run registries are considered to have no loss to follow-up. This study was approved by the Regional Ethical Review Board in Stockholm and the Swedish National Board of Health and Welfare (2011/136-31/5 and 2018/1591-31/2). According to the Swedish ethics board and General Data Protection Regulation legislation, informed consent is not required for pseudo-anonymized register-based research.

### Study Populations and Study Design

The study population includes patients with CKD enrolled in SRR from January 1, 2011 to December 31, 2021. Using the longitudinal information from the SRR and data on transition (i.e., progression to a more severe stage of CKD, start and/or switch dialysis modality or receiving a kidney transplant), we created the following 4 different cohorts of patients with: (i) CKD stage 4, (ii) CKD stage 5-ND, (iii) on maintenance HD, and (iv) on PD.^36^ Patients were censored at the time of CKD progression (i.e., when they progressed to a more severe CKD stage) or switch dialysis modality during the studied period. Within this study design, a given patient may contribute to more than one cohort if he/she progresses, enabling us to report event rates specific to each CKD stage and dialysis modality, as clinical profile differs when patients transition between stages and dialysis modalities. In each cohort, the index date was the date of the first recorded visit within the required CKD stage or dialysis modality. Patients were followed until the occurrence of HF, death, transition to a more severe CKD stage or change in dialysis modality, or on December 31, 2021, whichever came first. The study design and the flowchart are presented in Supplementary Figures S1 and S2.

A nationwide database including all individuals living in Sweden and born in 1960 or earlier^37^ served as the source for the control population. For each patient with CKD, 10 individuals were randomly selected from the GP, matched on sex and year of birth. The index date of each control individual was the same as that of the corresponding CKD patient. Some patients with CKD could not be matched because they were born after 1960 (16%), but were kept in the analyses for completeness.

### Covariates

The covariates were derived from the index date and included age, sex, comorbidities, ongoing medications (Supplementary Table S1). Comorbidities included hypertension, diabetes mellitus, history of ischemic heart disease, myocardial infarction, cerebrovascular disease, peripheral artery disease, and HF. Ongoing medications dispensed at any pharmacy within the 6 months before index date, included RASi, beta-blockers, calcium channel blockers, diuretics (thiazides and thiazides like diuretics, loop diuretics, mineralocorticoid receptor antagonists [MRA]), angiotensin receptor-neprilysin inhibitor, and sodium-glucose cotransporter 2 inhibitors.

### Outcomes

Study outcomes were time-to-first hospitalization for HF, defined as HF being the main reason for hospital admission, and identified through International Classification of Diseases-10 codes recorded in the National Patient Registry; and HF-related death, defined as the primary cause of death in the Cause of Death Registry. International Classification of Diseases codes for HF in Swedish registries have been validated against European Society of Cardiology definition in several independent studies, all demonstrating high diagnostic accuracy.^38^, ^39^, ^40^

As exploratory analysis, given that HF may result from different cardiovascular etiologies, we also examined the underlying cause of HF-related death, recorded in any diagnosis position (other than the primary) in the Cause of Death Registry. Underlying causes of HF-related death included the following: ischemic heart disease, arrythmia and sudden cardiac death, valvular heart disease, cardio-renal syndrome, and other CVD. Detailed definitions to defined outcomes are provided in Supplementary Table S1.

### Statistical Analyses

Baseline characteristics are presented by CKD stage and dialysis modality. Categorical variables are presented as frequency and percentage, and continuous variables are presented as mean (SD) or median (Q1-Q3, first-third quartile), as appropriate.

We conducted 2 different sets of analyses. In the first analysis, we reported event rates across CKD stage or dialysis modality, and sex. IR per 100 person-year (PY) for HF-related hospitalization and HF-related death were calculated using Poisson regression. To account for potential changes over time in event rates in comparison to those in the GP, we calculated standardized IRRs according to indirect standardization by taking the observed number of events divided by the expected number of events for CKD populations based on the observed rates in the age-, sex-matched GP, with 95% confidence intervals computed assuming a Poisson distribution.

In the second analysis, we examined temporal trends in HF hospitalization and HF mortality rates. Calendar years were divided into 2-year blocks, with the period 2011 to 2012 as the reference category. Changes in event rates and time periods were evaluated using 2 complimentary approaches—percentage changes from the reference period (2011-2012), and age-sex-adjusted incidence rate ratio (IRR) associated with calendar year. IRR were estimated using Poisson regression models with log-time as an offset variable to account for different follow-up durations. The *P*-value for trend was calculated considering calendar year as a continuous variable.

Analyses were stratified on sex and by the ongoing use of diuretic therapy. As sensitivity analysis, models were rerun as follows: (i) restricting the study population to matched CKD patients, that is, patients born before 1960; (ii) rematching the patients from the CKD cohorts with a new set of controls from the GP; and (iii) restricting the study population to patients without history of HF at baseline. Finally, in patients for whom HF was the primary cause of death, we described the underlying cause of HF, by identifying the first recorded CVD diagnosis following HF, based on the International Classification of Diseases-10 diagnosis coding.

Study covariates had no missing values. Statistical significance was defined as 2-tailed *P*-values < 0.05. The STROBE statement was followed for reporting observational studies.^41^ All statistical analyses were conducted using R software (version 4.4.1; R Project for Statistical Computing, Vienna, Austria).

## Results

### Baseline Characteristics

Between 2011 and 2021, a total of 25,591 patients with CKD stage 4 (mean age 71 years; 36.5% women), 13,968 with CKD stage 5-ND (age 69 years; 36.9% women), 10,635 receiving HD (age 65 years; 34.2% women) and 4511 patients receiving PD (age 64 years; 31.9% women) were identified in the SRR (Table 1). At baseline, CVD was common across all groups, with the highest prevalence among patients on HD. HF was the most frequent CVD, with a prevalence of 27%, 25%, 33%, and 25% in CKD stage 4, 5-ND, HD, PD, respectively. RASi use was lower across CKD cohorts, whereas beta-blocker use increased. Finally, although loop diuretics use also showed a progressive increase across CKD cohorts, other diuretic classes decreased.

**Table 1:** Characteristics of the population by CKD stage and dialysis modality at time of registry enrollment

Characteristics	CKD stage 4	CKD stage 5-ND	Hemodialysis	Peritoneal dialysis
	*n* = 25,591	*n* = 13,968	*n* = 10,635	*n* = 4511
Clinical and biological data				
Age, yrs	70.5 (13.6)	69.2 (14.8)	65.0 (15.1)	63.6 (15.8)
Sex, Women	9335 (36.5)	5149 (36.9)	3634 (34.2)	1440 (31.9)
Systolic blood pressure, mm Hg	139 (21.5)	141 (21.3)	144 (24.4)	137 (20.6)
Diastolic blood pressure, mm Hg	76 (11.9)	78 (12.1)	75 (15.7)	77 (12.3)
Body mass index, kg/m^2^	28.2 (5.8)	27.8 (5.9)	26.4 (5.8)	26.2 (4.7)
eGFR, ml/min per 1.73 m^2^	23 (4.2)	12 (2.3)	-	-
Albuminuria, mg/mmol (Q1–Q3)	28.1 (5.4, 127.0)	94.2 (23.2, 237.0)	-	-
Medical history				
Hypertension	24713 (96.6)	13573 (97.2)	10256 (96.4)	4466 (99.0)
Diabetes mellitus	11206 (43.8)	5846 (41.9)	4780 (44.9)	1786 (39.6)
Any cardiovascular disease	9766 (38.2)	5049 (36.1)	4510 (42.4)	1598 (35.4)
Ischemic heart disease	7501 (29.3)	3686 (26.4)	3304 (31.1)	1220 (27.0)
Myocardial infarction	5480 (21.4)	2680 (19.2)	2437 (22.9)	887 (19.7)
Cerebrovascular disease	4340 (17.0)	2352 (16.8)	1911 (18.0)	718 (15.9)
Peripheral artery disease	3158 (12.3)	1628 (11.7)	2006 (18.9)	580 (12.9)
Heart failure	6882 (26.9)	3554 (25.4)	3498 (32.9)	1138 (25.2)
Ongoing medications				
Any antihypertensive therapy	24196 (94.5)	13338 (95.5)	9811 (92.3)	4390 (97.3)
Renin-angiotensin system inhibitor	17655 (69.0)	8579 (61.4)	4981 (46.8)	2351 (52.1)
Calcium channel blocker	14837 (58.0)	9623 (68.9)	6454 (60.7)	3163 (70.1)
Beta-blocker	16531 (64.6)	9496 (68.0)	7664 (72.1)	3377 (74.9)
Diuretic therapy	16629 (65.0)	9912 (71.0)	7784 (73.2)	3865 (85.7)
Thiazide & thiazide-like	3917 (15.3)	1252 (9.0)	467 (4.4)	301 (6.7)
Loop diuretic	15473 (60.5)	9613 (68.8)	5898 (55.5)	3291 (73.3)
MRA	2382 (9.3)	846 (6.1)	393 (3.7)	217 (4.8)
Amiloride	287 (1.1)	70 (0.5)	8 (0.1)	9 (0.2)
ARNI	119 (0.5)	21 (0.2)	13 (0.1)	6 (0.1)
SGLT2 inhibitors	157 (0.6)	29 (0.2)	10 (0.1)	3 (0.1)
Dialysis characteristics				
Kt/V	-	-	2.1 (0.5)	2.2 (0.6)
Dialysis modality				
Hemodialysis	-	-	7165 (67.4)	-
Hemodiafiltration	-	-	3241 (30.5)	-
Hemofiltration	-	-	24 (0.2)	-
Home hemodialysis	-	-	205 (1.9)	-
Automated PD (APD)	-	-	-	1336 (29.6)
Continuous ambulatory PD (CAPD)	-	-	-	3175 (70.4)
Dialysis access				
Catheter (temporary/tunneled)	-	-	5452 (51.3)	-
AV-fistula	-	-	4456 (41.9)	-
Graft	-	-	699 (6.6)	-
Other vascular accesses	-	-	23 (0.2)	-
PD catheter	-	-	-	4511 (100.0)

ARNI, angiotensin receptor-neprilysin inhibitor; CKD, chronic kidney disease; eGFR, estimated glomerular filtration rate; HF, heart failure; MRA, mineralocorticoid receptor antagonist; ND, nondialysis; SGLT2 inhibitor, sodium-glucose cotransporter-2 inhibitor.

Continuous variables are presented as mean (SD) or median (Q1–Q3), as appropriate. Categorical variables are presented as number (percentage).

### IR of HF by CKD Stage and Dialysis Modality, Compared With the GP

The IR of HF-related hospitalization was higher in ND-CKD (IR: 6.8 and 9.5 per 100-PY in CKD stage 4 and stage 5-ND, respectively) than in patients on maintenance dialysis (5.2 and 5.4 per 100-PY in HD and PD, respectively), higher in men than in women, and 3.7 to 6.8-fold higher than an age-sex-matched GP (Table 2).

**Table 2:** Incidence rates of HF outcomes, by CKD cohort and sex

Population	HF-related hospitalization		Death because of HF	
	*N* events	IR per 100-PY (95%CI)	*N* events	IR per 100-PY (95%CI)
General population				
Overall	32118 / 443717	1.26 (1.24–1.27)	5813 / 443717	0.22 (0.22–0.23)
Men	22903 / 286375	1.42 (1.40–1.44)	3844 / 286375	0.23 (0.22–0.24)
Women	9215 / 157342	0.98 (0.96–1.00)	1969 / 157342	0.20 (0.19–0.21)
CKD stage 4				
Overall	4434 / 25591	6.84 (6.64–7.05)	1816 / 25591	2.59 (2.48–2.72)
Men	2903 / 16256	7.42 (7.16–7.70)	1223 / 16256	2.89 (2.73–3.05)
Women	1531 / 9335	5.96 (5.67–6.27)	593 / 9335	2.14 (1.98–2.32)
CKD stage 5				
Overall	1938 / 13968	9.52 (9.10–9.95)	811 / 13968	3.74 (3.49–4.00)
Men	1183 / 8819	9.97 ( 9.41–10.5)	485 / 8819	3.86 (3.53–4.22)
Women	755 / 5149	8.89 (8.27–9.54)	326 / 5149	3.57 (3.20–3.97)
Hemodialysis				
Overall	1349 / 10635	5.18 (4.91–5.46)	717 / 10635	2.55 (2.37–2.74)
Men	920 / 7001	5.49 (5.14–5.85)	481 / 7001	2.64 (2.41–2.88)
Women	429 / 3634	4.63 (4.21–5.09)	236 / 3634	2.37 (2.08–2.69)
Peritoneal dialysis				
Overall	349 / 4511	5.44 (4.89–6.03)	172 / 4511	2.57 (2.20–2.97)
Men	241 / 3071	5.51 (4.84–6.23)	127 / 3071	2.79 (2.33–3.30)
Women	108 / 1440	5.29 (4.36–6.36)	45 / 1440	2.10 (1.55–2.78)

CKD, chronic kidney disease; HF, heart failure; PY, person years.

The IR of HF-related death was similarly high in patients with CKD stage 4 as in those on dialysis (IR: 2.6 per 100-PY), and the highest among those with CKD stage 5-ND (IR: 3.7 per 100-PY), representing 11.6 to 17.0-fold higher rate compared with the age-sex-matched GP (Table 2). The main cause of HF-related deaths was cardio-renal syndrome (64 to 71%), followed by ischemic heart disease (16.5– 21.5%), arrythmia and/or sudden cardiac death (6.5–10%), and valvular disease (4–5.2%), and other CVDs (1.2–2.4%) (Supplementary Figure S3).

### Patient Characteristics Over Time

**Figure 1:**
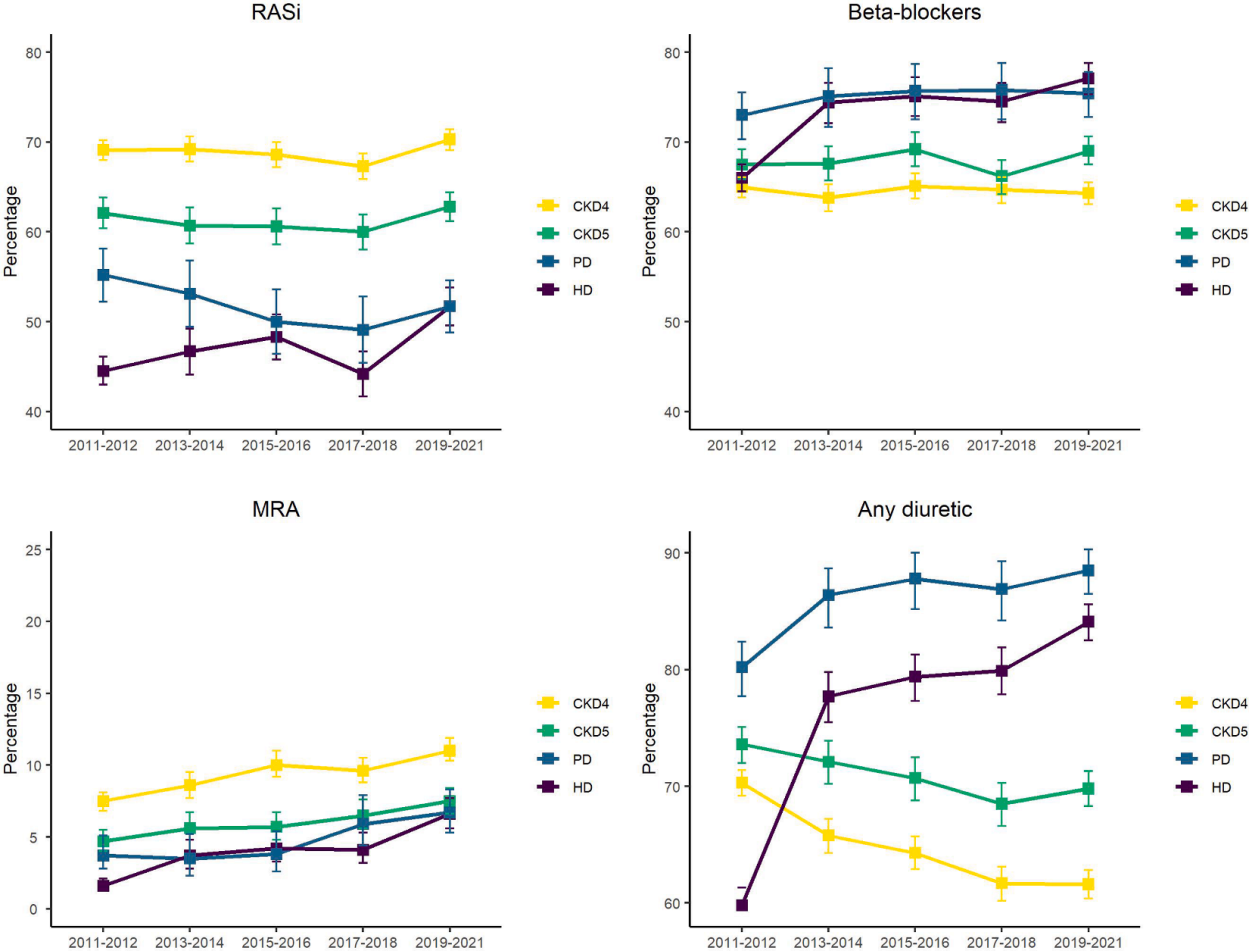
**Prevalence of HF medications by CKD cohort and calendar year**. Error bars indicate 95% confidence intervals. CKD, chronic kidney disease; HD, hemodialysis; MRA, mineralocorticoid receptor antagonist; PD, peritoneal dialysis; RASi, renin-angiotensin system inhibitor.

**Figure 2:**
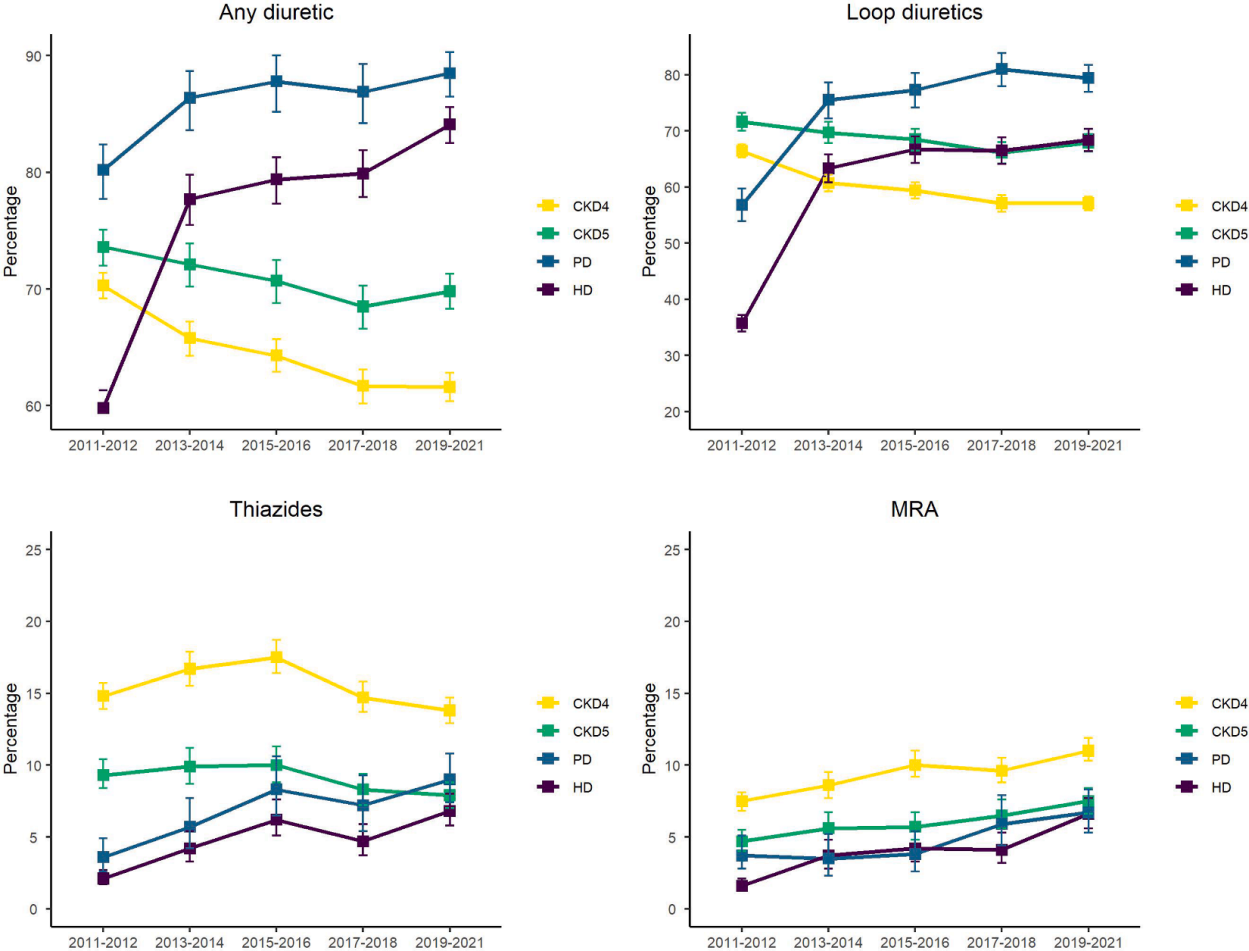
**Prevalence of diuretic medications by CKD cohort and calendar year**. Error bars indicate 95% confidence intervals. CKD, chronic kidney disease; HD, hemodialysis; MRA, mineralocorticoid receptor antagonist; PD, peritoneal dialysis.

**Figure 3:**
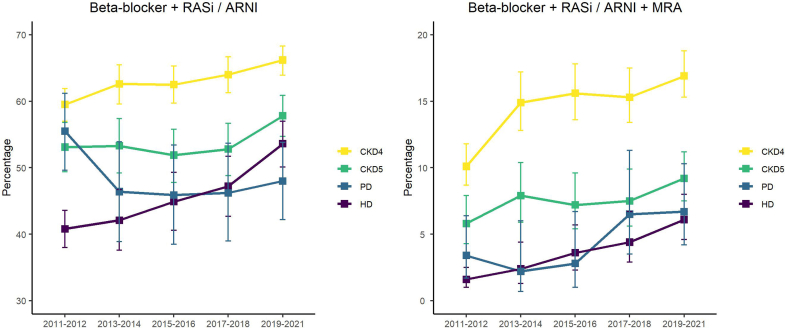
**Prevalence of bi- and tritherapy in patients with HF at baseline, by CKD cohort and calendar year**. Error bars indicate 95% confidence intervals. ARNI, angiotensin receptor-neprilysin inhibitor; CKD, chronic kidney disease; HD, hemodialysis; MRA, mineralocorticoid receptor antagonist; PD, peritoneal dialysis; RASi, renin-angiotensin system inhibitor.

Baseline characteristics of incident patients are presented by 2-year calendar blocks in Supplementary Tables S2–S5. Between 2011 and 2021, the prevalence of diabetes and body mass index (up to + 0.8 kg/m^2^) increased in all CKD cohorts. In contrast, although the proportion of patients with CVD remained relatively stable in both ND-CKD cohorts, it decreased in dialysis patients. The prevalence of HF increased across all CKD cohorts, except in patients on PD, where it remained stable across calendar years. The medical treatment pattern also varied over time (Figures 1 and 2). Although the use of RASi remained low and stable in all cohorts, the proportion of patients receiving beta-blockers increased only in patients on dialysis. Loop diuretics use declined in ND-CKD, but rose substantially in patients on dialysis, particularly in HD. In contrast, although the use of MRA remained low, it steadily increased across all CKD cohorts. In patients with HF at baseline, the proportion receiving tritherapy (beta-blocker + RASi/ARNi + MRA) remained low (2%–10%), but gradually increased in all cohorts over time, reaching 6% to 16% by 2021 (Figure 3). In CKD stage 4, men were more frequently prescribed RASi and beta-blockers, whereas women were slightly more often prescribed MRAs and diuretics. These differences attenuated with advancing CKD, with no major sex differences observed in stage 5 or in dialysis patients (Supplementary Figures S4 and S5).

### Temporal Trends in the Rates of HF Outcomes by CKD Stages

**Figure 4:**
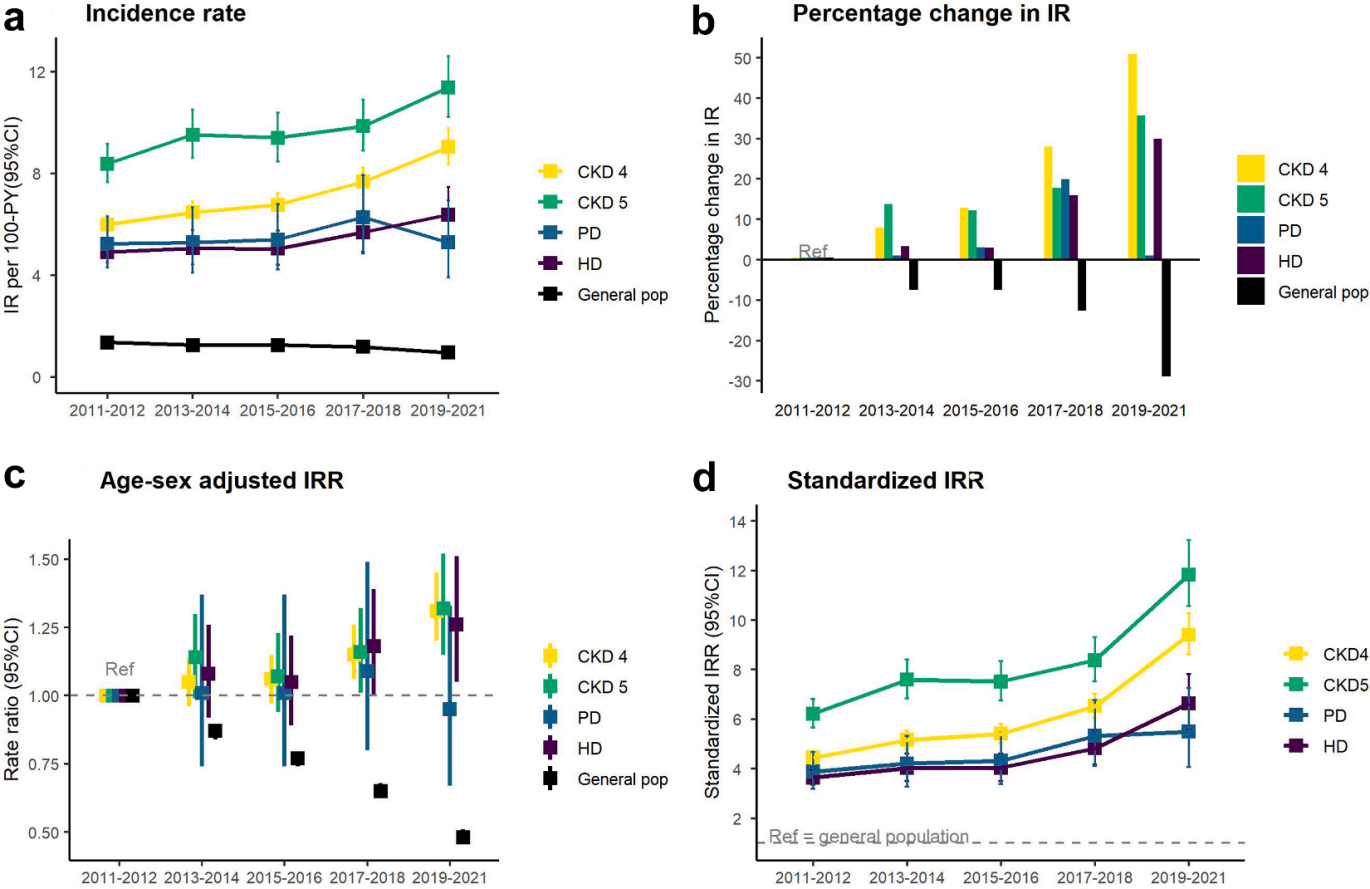
**Incidence rates (IR) and adjusted incidence rate ratios (IRR) ofHF-relatedhospitalization by calendar year and CKD cohort**. (a) IR. (b) Percentage change in IR compared with 2011-2012. A negative value indicates a decrease in rate, whereas a positive value indicates an increase in rate of HF. (c) IRR were estimated using Poisson regression, adjusted for age and sex. (d) Standardized IRR reflects relative risk compared with a 1:10 age- and sex-matched GP. A steeper decline in IR in the GP derives the increase in standardized IRR. Standardized IRRs were calculated according to indirect standardization by taking the observed number of events divided by the expected number of events for CKD populations based on the observed rates in the age-, sex- matched GP, with 95% confidence intervals computed assuming a Poisson distribution. CI, confidence intervals; GP, general population; IR, incidence rate; IRR, incidence rate ratio.

Over time, HF-related hospitalizations increased in all CKD cohorts, as much as 51% and 36% in CKD stage 4 and 5-ND, and 30% in HD. In PD, it increased by 20% in 2017 to 2019 before decreasing in 2019 to 2021 (Figure 4, Supplementary Table S6). Compared with 2011 and 2012, age-sex adjusted IRR showed a gradual increase across subsequent years in all cohorts (adjusted IRR: 1.31 in CKD stage 4, 1.32 in CKD stage 5-ND, 1.26 in HD), except in PD where adjusted IRR decreased (adjusted IRR 0.71 [0.54–0.93] in 2019–2021) (Figure 4, Supplementary Table S6). This contrasts with the gradual decrease observed in the GP. By 2021, IR of HF-related hospitalization in CKD reached 5.5 to 11.8-fold higher than that in the GP. In general, similar patterns were observed after exclusion of nonmatched patients with CKD (Supplementary Table S9), after rematching patients with CKD with another set of controls from the GP (Supplementary Table S11), in patients without history of HF (Supplementary Table S12), and across strata of sex (Supplementary Table S14) and diuretic use (Supplementary Table S15). Of note, rates were higher in men than in women, although sex-differences narrowed after dialysis start.

**Figure 5:**
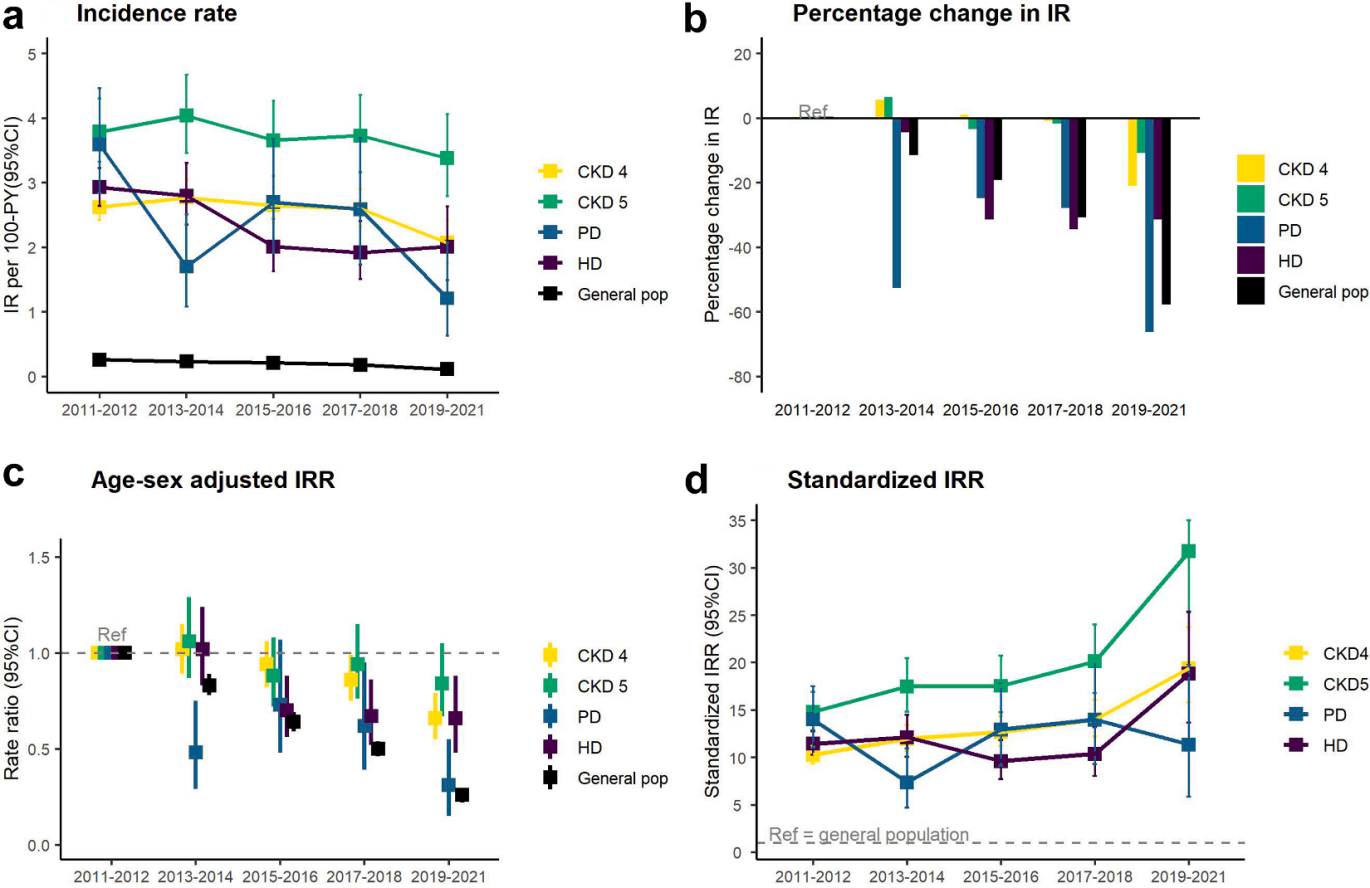
**Incidence rates (IR) and adjusted incidence rate ratios (IRR) of death because of HF, by calendar year and CKD cohort**. (a) IR. (b) Percentage change in IR compared with 2011-2012. A negative value indicates a decrease in rate, whereas a positive value indicates an increase in rate of HF-related death. (c) IRR were estimated using Poisson regression, adjusted for age and sex. (d) Standardized IRR reflects relative risk compared with a 1:10 age- and sex-matched GP. A steeper decline in IR in the GP derives the increase in standardized IRR. Standardized IRRs were calculated according to indirect standardization by taking the observed number of events divided by the expected number of events for CKD populations based on the observed rates in the age-, sex- matched GP, with 95% confidence intervals computed assuming a Poisson distribution. CI, confidence intervals; GP, general population; IR, incidence rate; IRR, incidence rate ratio.

In contrast, the HF mortality rate steadily decreased both in ND-CKD (up to 21% in CKD stage 4) and in dialysis dependent CKD (up to 66% among patients on PD) (Figure 5, Supplementary Table S7). Compared with 2011 and 2012, age-sex adjusted IRR showed a gradual decrease in HF-related death across all CKD cohorts – from 0.31 in PD, 0.66 in CKD stage 4 and HD, and 0.84 in CKD stage 5-ND. However, the observed decline was in general much slower than that observed in the age-sex matched GP (Figure 5, Supplementary Table S7). By 2021, IR of death because of HF was 11.4 to 32 fold higher than that in the age-sex matched population. The rates of all-cause mortality also decreased both in ND-CKD (up to -6.8% in CKD 5) and in dialysis-dependent CKD (-28.9%), but in a lower magnitude than death because of HF (Supplementary Table S8). Similar results were observed after exclusion of nonmatched CKD patients (Supplementary Table S9) and after rematching patients with CKD with another set of controls from the GP (Supplementary Table S11) and in patients without history of HF (Supplementary Table S13).

## Discussion

In this nationwide cohort study of patients with advanced CKD, we provide a comprehensive and contemporary assessment of HF-related hospitalization and HF-related death, as well as temporal trends between 2011 and 2021. We observed different trends in HF-related hospitalization and HF-related death in patients with CKD of different stages and dialysis modalities, and in comparison with the GP. Specifically, the rate of HF-related hospitalization was relatively high and substantially increased, particularly among patients with ND-CKD and on HD, contrary to the gradual decrease observed in the GP. In contrast, during the same time period, HF-related death decreased in all CKD cohorts despite the changing patients’ clinical profiles, although improvement remained much slower than in the GP. Our results were robust across subgroup and sensitivity analyses. This emphasizes the growing excess cardiovascular burden in patients with CKD and the need for optimized evidence-based treatment strategies tailored to this populations, as well as more consistent implementation of guideline-directed medical therapy to reduce HF-related morbidity.

### Rates in HF Outcomes

Our study provides new and contemporary insight into the epidemiology of HF in patients with advanced CKD, and capture the changing landscape of novel cardio-renal therapies, offering valuable information for policy makers, trialists, and health resource planners. In our study, we observed that rates of HF-related hospitalization and HF-related death were high—overall respectively 3.7 to 6.8 times (HF hospitalization) and 11.6 to 17.0-fold (HF death) higher than in the age-sex-matched GP—and higher in patients with ND-CKD compared with those on dialysis.^3^ Rates were also higher in men than in women, although sex-differences narrowed after dialysis start.

### Temporal Trends in HF Outcomes

Although absolute numbers and relative changes in HF rates are clinically relevant, (standardized) temporal trends are essential for monitoring disease over time, for comparing disease burden across populations, for evaluating treatment impact, and for guiding healthcare resource planning. Our study provides temporal trends in patients with advanced CKD in comparison with the GP, expanding evidence from older periods.^29^^,^^30^ Previous studies reported a high prevalence of HF among CKD patients, and conversely, a high prevalence of CKD among patients with HF. However, data on temporal trends in HF-related hospitalization and death because of HF in patients with CKD remain limited over the last 2 decades,^29^^,^^30^ focusing on cardiovascular mortality^29^^,^^42^ and only considered patients with kidney failure.^29^^,^^30^^,^^42^ In a population-based cohort of 926,298 US dialysis patients from the US Renal Data System, Stack *et* al.^30^ showed that, although the prevalence of HF at dialysis start remained relatively stable, HF-related mortality declined between 1995 and 2005. Similarly, a study conducted in the Australia and New Zealand Dialysis and Transplant registry reported a decrease in cardiovascular mortality rate among 34,741 prevalent patients on dialysis (64% HD, 36% PD) between 1992 and 2005, whereas excess cardiovascular risk compared with the GP increased during the same period.^29^ Finally, a recent analysis of about 1.9 million US death certificates of patients with a clinical diagnosis of CKD showed that, between 1999 and 2020, there was a 7.1% decrease in cardiovascular mortality, whereas the age-adjusted all-cause mortality increased by 50% over the same period.^42^

In our study, we observed different patterns in temporal trends across HF outcomes. Although rates of HF-related hospitalization steadily increased—particularly in patients with ND-CKD and those on HD—HF-related mortality gradually declined. Nonetheless, these opposite trends translated into a growing excess risk of both HF-related hospitalization and mortality as compared with the GP. Over the same period, we observed that the prevalence of comorbid conditions, such as diabetes, obesity, and CVD also rose, as previously reported in HF population.^24^ Although progresses in clinical management of acute episodes of HF may have contributed to decrease HF-related mortality, increasing rates of HF-related hospitalization suggest suboptimal implementation of guideline-recommended therapies in this population, possibly because of concerns about potential adverse events and uncertainty regarding efficacy, as clinical trials have mainly focused on non-CKD populations.^15^^,^^16^ For instance, the use of RASi was particularly low, and diuretic use decreased by 5% to 10% in patients with ND-CKD—a population in which rates of HF-related hospitalization concomitantly increased by 36% to 51%. Among patients with known HF at baseline, a bitherapy (beta-blocker + RASi/ARNi) was prescribed in 40% to 60% of patients, whereas the tritherapy (beta-blocker + RASi/ARNi + MRA) was used in only 2% to 10% of patients, evidencing a room for improvement. Further benefits may also be expected with the ongoing wider implementation of novel cardio- and nephro-protective therapies like sodium-glucose cotransporter 2 inhibitors,^43^ selective nonsteroidal mineralocorticoid receptor antagonists,^44^ or glucagon-like peptide-1 receptor agonists,^45^ when indicated.

Other contributing factors may also explain the observed trends. The rising rates of HF in this relatively old population may reflect suboptimal control of cardiovascular risk factors and the underutilization of HF-modifying therapies,^7^^,^^11^ whereas a higher burden of comorbidities may have lowered the threshold for hospital admissions. Moreover, improved management of acute HF episodes may have contributed to better survival rates, despite an increase in hospital admissions. In addition, policy interventions aiming at promoting earlier diagnosis, improved access to healthcare, change in coding practices, and the development of specialized cardio-renal units may have contributed to increased HF diagnoses over time. Differences observed between patients on HD and PD might be explained by the decrease in the frequency of HD sessions, alongside with daily exchanges and a larger use of Icodextrin in PD.^46^

### Strengths and Limitations

Our study has several strengths including the complete national capture of well-characterized patients referred to nephrology care in Sweden, the evaluation of HF-specific outcomes across specific stages of CKD severity and dialysis modalities, no loss to follow-up thanks to the linkage with Government-run registries, the standardization of event rates relative to the GP, and the setting of Swedish universal tax-funded healthcare which avoids selection bias from disparate access to healthcare or disaggregated data sources. However, our study has also some limitations. First, as in all observational studies based on electronic medical records, event rates reflect patients with a clinical diagnosis that was documented in their medical records. Differentiating hospitalization for fluid overload because of “true” cardiac dysfunction from advanced kidney disease is sometime challenging^47^ and diagnosis of all “HF-related” hospitalizations should be ideally confirmed by an echocardiography. Furthermore, the recording and the positioning of clinical diagnoses may be influenced by coding practices and reimbursement, which could have introduce bias or misclassification. However, there is no reason to believe that coding practice would differ between patients with CKD and the GP, and only HF as the main diagnosis was considered. Second, HF-related hospitalization may have been underestimated during the COVID-19 pandemic, as frail patients with advanced CKD might have been less willing to be hospitalized for a health problem other than a COVID-19 infection, and overlapping clinical symptoms between HF and COVID-19 pneumonia could have led to misclassification. Third, information on ejection fraction or biomarkers such as brain natriuretic peptides were not available in our database, and International Classification of Diseases codes separating HF with reduced ejection fraction and HF with preserved ejection fraction were introduced in 2021 in Sweden. Fourth, data on adherence were not available, but capturing dispensed (rather than prescribed) medications provides a closer proxy for actual use. In addition, the study period predated the widespread availability and indications for sodium-glucose cotransporter2 inhibitor use in CKD, and future studies are needed to assess HF trends after their introduction. Finally, although previous studies have shown that patients with CKD from the SRR are comparable to those reported in European cohorts from the European Renal Association registry supporting its external validity for European CKD populations,^33^ extrapolation of our findings to other ethnicities (the Swedish population being predominantly of Caucasian origin) with earlier stages of CKD should be undertaken with caution, particularly in the absence of supporting evidence from previous studies.

### Conclusion

In conclusion, results from this large nationwide cohort of patients with advanced CKD, we observed that, despite a decline in HF-related mortality—although slower than that in the GP—rates of HF-related hospitalization increased substantially between 2011 and 2021, as compared with the general improvement seen in the age-sex-matched GP. These findings emphasize the growing excess cardiovascular burden in patients with advanced CKD, and underscore the urgent need for optimized evidence-based treatment strategies tailored to this very high-risk populations, as well as more consistent implementation of guideline-directed medical therapy to reduce HF-related morbidity.

## Disclosure

All the authors declared no competing interests. Unrelated to the study, ME reports personal honoraria for lectures by AstraZeneca, Astellas pharma, Vifor Pharma, Fresenius healthcare, Baxter Healthcare, and being member of advisory boards for Astellas, AstraZeneca, and Vifor Pharma. J-JC reports funding to Karolinska Institutet by AstraZeneca, Astellas, Amgen, Vifor Pharma, Novo Nordisk, and MSD; personal honoraria for lectures by Fresenius Kabi, Baxter Healthcare and Abbott, and being member of advisory boards for Astellas, AstraZeneca, and GSK.

## Supplementary Material

Supplementary file (PDF) **Figure S1.** Study design. **Figure S2.** Flowchart for the study population. **Figure S3.** Underlying cause of HF-related death, by CKD cohort, in patients with HF as the main cause of death. **Figure S4.** Prevalence of HF medications by sex and calendar year, in nondialysis CKD. **Figure S5.** Prevalence of HF medications by sex and calendar year, in dialysis CKD. **Table S1.** Definitions of comorbidities, medications and outcomes. **Table S2.** Baseline characteristics by calendar years, in patients with CKD stage 4. **Table S3.** Baseline characteristics, by calendar year, in patients with CKD stage 5-ND. **Table S4.** Baseline characteristics by calendar year, in patients on hemodialysis. **Table S5.** Baseline characteristics by calendar year, in patients on peritoneal dialysis. **Table S6.** Number of events, incidence rates per 100-PY, age-sex adjusted incidence rate ratios of heart failure-related hospitalization, by calendar year. **Table S7.** Number of events, incidence rates per 100-PY, age-sex adjusted incidence rate ratios of death due to heart failure, by calendar year. **Table S8.** Number of events, incidence rates per 100-PY, age-sex adjusted incidence rate ratios of all-cause mortality, by calendar year. **Table S9.** Sensitivity analyses (exclusion of nonmatched CKD patients): incidence rates and standardized incidence rate ratios of heart failure-related hospitalization. **Table S10.** Sensitivity analyses (exclusion of nonmatched CKD patients): incidence rates and standardized incidence rate ratios of death because of heart failure. **Table S11.** Sensitivity analyses: standardized incidence rate ratios of HF-related hospitalization and death due to HF, after rematching patients with CKD with another set of controls from the general population. **Table S12.** Sensitivity analyses: standardized incidence rate ratios of heart failure-related hospitalization and, in patients without history of heart failure. **Table S13.** Sensitivity analyses: standardized incidence rate ratios of death due to heart failure, in patients without history of heart failure. **Table S14.** Number of events, incidence rates and age adjusted incidence rate ratios of heart failure-related hospitalization, by CKD cohort, calendar year and sex. **Table S15.** Number of events, incidence rates and age adjusted incidence rate ratios of heart failure-related hospitalization, by CKD cohort, calendar year and diuretic use.

## Footnotes

Supplementary file (PDF)
**Figure S1.** Study design. **Figure S2.** Flowchart for the study population. **Figure S3.** Underlying cause of HF-related death, by CKD cohort, in patients with HF as the main cause of death. **Figure S4.** Prevalence of HF medications by sex and calendar year, in nondialysis CKD. **Figure S5.** Prevalence of HF medications by sex and calendar year, in dialysis CKD. **Table S1.** Definitions of comorbidities, medications, and outcomes. **Table S2.** Baseline characteristics by calendar years, in patients with CKD stage 4. **Table S3.** Baseline characteristics, by calendar year, in patients with CKD stage 5-ND. **Table S4.** Baseline characteristics by calendar year, in patients on hemodialysis. **Table S5.** Baseline characteristics by calendar year, in patients on peritoneal dialysis. **Table S6.** Number of events, incidence rates per 100-PY, age-sex adjusted incidence rate ratios of heart failure-related hospitalization, by calendar year. **Table S7.** Number of events, incidence rates per 100-PY, age-sex adjusted incidence rate ratios of death due to heart failure, by calendar year. **Table S8.** Number of events, incidence rates per 100-PY, age-sex adjusted incidence rate ratios of all-cause mortality, by calendar year. **Table S9.** Sensitivity analyses (exclusion of nonmatched CKD patients): incidence rates and standardized incidence rate ratios of heart failure-related hospitalization. **Table S10.** Sensitivity analyses (exclusion of nonmatched CKD patients): incidence rates and standardized incidence rate ratios of death because of heart failure. **Table S11.** Sensitivity analyses: standardized incidence rate ratios of HF-related hospitalization and death due to HF, after rematching patients with CKD with another set of controls from the general population. **Table S12.** Sensitivity analyses: standardized incidence rate ratios of heart failure-related hospitalization and, in patients without history of heart failure. **Table S13.** Sensitivity analyses: standardized incidence rate ratios of death due to heart failure, in patients without history of heart failure. **Table S14.** Number of events, incidence rates and age adjusted incidence rate ratios of heart failure-related hospitalization, by CKD cohort, calendar year and sex. **Table S15.** Number of events, incidence rates and age adjusted incidence rate ratios of heart failure-related hospitalization, by CKD cohort, calendar year and diuretic use. STROBE Checklist.
