# Supplementary material for: 10-Year Trends in Heart Failure Among Patients With Dialysis and Nondialysis CKD
Source: Kidney Int Rep. 2026 Jul 4;11(9):106695. doi: 10.1016/j.ekir.2026.106695 (PMC13453444; doi:10.1016/j.ekir.2026.106695)
Supplement: Supplementary file 1 [file mmc1.pdf]

SUPPLEMENTARY MATERIAL

10-year trends in heart failure in patients with CKD stages 4-5 and dialysis: a nationwide cohort study

Roosa Lankinen, M.D, Ph.D[1], Shunsuke Murata, Ph.D[2,3,4], Juan-Jesús Carrero, Pharm.D, Ph.D[1,5], Karin Modig, Ph.D[2], Marie Evans, M.D, Ph.D[6,7], Anne-Laure Faucon, M.D, Ph.D[1,6,7]

1. Department of Medical Epidemiology and Biostatistics, Karolinska Institutet, Stockholm, Sweden
2. Unit of Epidemiology, Institute of Environmental Medicine, Karolinska Institutet, Stockholm, Sweden
3. Frontier Research Institute for Interdisciplinary Sciences, Tohoku University, Sendai, Japan
4. Department of International and Community Oral Health, Tohoku University Graduate School of Dentistry, Sendai, Japan
5. Department of Clinical Sciences, Danderyd Hospital, Stockholm, Sweden
6. Department of Nephrology, Karolinska University Hospital, Stockholm, Sweden.
7. Department of Clinical Science, Intervention and Technology, Karolinska Institutet, Stockholm, Sweden

TABLE OF CONTENTS

Table S1. Definitions of comorbidities, medications and outcomes 3

Table S2. Baseline characteristics by calendar years, in patients with CKD stage 4 4

Table S3. Baseline characteristics, by calendar year, in patients with CKD stage 5-ND..................... 5

Table S4. Baseline characteristics by calendar year, in patients on hemodialysis 6

Table S5. Baseline characteristics by calendar year, in patients on peritoneal dialysis 7

Table S6. Number of events, incidence rates per 100-PY, age-sex adjusted incidence rate ratios of heart failure-related hospitalization, by calendar year.. 8

Table S7. Number of events, incidence rates per 100-PY, age-sex adjusted incidence rate ratios of death due to heart failure, by calendar year.. 9

Table S8. Number of events, incidence rates per 100-PY, age-sex adjusted incidence rate ratios of all-cause mortality, by calendar year.. 10

Table S9. Sensitivity analyses (exclusion of non-matched CKD patients): incidence rates and standardized incidence rate ratios of heart failure-related hospitalization. 11

Table S10. Sensitivity analyses (exclusion of non-matched CKD patients): incidence rates and standardized incidence rate ratios of death due to heart failure. 12

Table S11. Sensitivity analyses: standardized incidence rate ratios of HF-related hospitalization and death due to HF, after re-matching CKD patients with another set of controls from the general population 13

Table S12. Sensitivity analyses: standardized incidence rate ratios of heart failure-related hospitalization and, in patients without history of heart failure.. 14

Table S13. Sensitivity analyses: standardized incidence rate ratios of death due to heart failure, in patients without history of heart failure 15

Table S14. Number of events, incidence rates and age adjusted incidence rate ratios of heart failure-related hospitalization, by CKD cohort, calendar year and sex. 16

Table S15. Number of events, incidence rates and age adjusted incidence rate ratios of heart failure-related hospitalization, by CKD cohort, calendar year and diuretic use. 17

Figure S1. Study design 18

Figure S2. Flowchart for the study population 19

Figure S3. Underlying cause of heart failure-related death, by CKD cohort, in patients with HF as the main cause of death 20

Figure S4. Prevalence of heart failure medications by sex and calendar year, in non-dialysis CKD. 21

Figure S5. Prevalence of heart failure medications by sex and calendar year, in dialysis CKD. 22

STROBE Statement 23

Table S1. Definitions of comorbidities, medications and outcomes

	ICD-10 codes	ATC codes
**Comorbidities**		
Diabetes mellitus	E10-E14	A10
Hypertension	I10-I15	C07, C09A, C09B, C09C, C09D, C08, C03A, C03B, C03C, C02A, C02C, C02D
Myocardial infarction	I21, I22, I252, I200	
Coronary artery disease	I20-I25	
Cerebrovascular disease	I60, I61, I62, I63, I64, I65, I66, I67, I69, G45, G46	
Peripheral artery disease	I70, I72, I73, I74	
Heart failure	I110, I130, I132, I50, J81	
**Medications**		
Renin-angiotensin system inhibitors		C09A, C09B, C09C, C09D
Calcium-channel blockers		C08
Beta-blockers		C07
Diuretics		C03
**Outcomes**		
Heart failure-related hospitalization	I110, I130, I132, I50, J81 as the main cause of hospitalization in the National Patient Registry	
Death due to heart failure	I110, I130, I132, I50, J81 as the main cause of death in the Cause of Death registry	
Heart failure etiologies	Diagnoses in any position other than primary	
Cardio-renal syndrome	I13, J81, I50	
Ischemic heart disease	I20-I25	
Valvular disease	I05, I06, I07, I08, I34, I35, I36, I37	
Arrythmia	I47-I49	
Cardiomyopathy	I42, I43	
Myocarditis	I40, I41, I51.5, I51.4	
Sudden death	I46	

For comorbidities, diagnoses in any positions were considered. ATC: anatomical therapeutic chemical classification, ICD-10: international classification of diseases 10th revision.

Table S2. Baseline characteristics by calendar years, in patients with CKD stage 4

CKD 4	2011-2012<br>N = 6574	2013-2014<br>N = 4050	2015-2016<br>N = 4440	2017-2018<br>N = 4436	2019-2021<br>N = 6091
**Clinical and biological data**					
Age, years	72 [63, 80]	73 [63, 80]	73 [64, 80]	74 [65, 80]	75 [66, 80]
Sex, Women	2356 (35.8)	1519 (37.5)	1667 (37.5)	1585 (35.7)	2208 (36.3)
Systolic blood pressure, mmHg	138 (21)	139 (22)	139 (22)	139 (22)	139 (22)
Diastolic blood pressure, mmHg	77 (12)	77 (12)	76 (12)	76 (12)	76 (12)
Body mass index, kg/m²	28.0 (5.5)	28.2 (5.7)	28.3 (5.9)	28.3 (6.0)	28.5 (6.0)
eGFR, mL/min per 1.73 m²	22 (4)	23 (4)	23 (4)	24 (4)	24 (4)
**Medical history**					
Hypertension	6372 (96.9)	3909 (96.5)	4289 (96.6)	4263 (96.1)	5880 (96.5)
Diabetes mellitus	2654 (40.4)	1743 (43.0)	1956 (44.1)	1999 (45.1)	2854 (46.9)
Any cardiovascular disease	2429 (36.9)	1572 (38.8)	1705 (38.4)	1743 (39.3)	2317 (38.0)
Ischemic heart disease	1960 (29.8)	1213 (30.0)	1300 (29.3)	1311 (29.6)	1717 (28.2)
Myocardial infarction	1403 (21.3)	863 (21.3)	959 (21.6)	978 (22.0)	1277 (21.0)
Cerebrovascular disease	1030 (15.7)	680 (16.8)	781 (17.6)	771 (17.4)	1078 (17.7)
Peripheral artery disease	842 (12.8)	546 (13.5)	511 (11.5)	563 (12.7)	696 (11.4)
Heart failure	1606 (24.4)	1060 (26.2)	1195 (26.9)	1239 (27.9)	1782 (29.3)
Atrial fibrillation / flutter	1217 (18.5)	843 (20.8)	991 (22.3)	1012 (22.8)	1515 (24.9)
**Ongoing medications**					
Any antihypertensive therapy	6260 (95.2)	3817 (94.2)	4216 (95.0)	4164 (93.9)	5739 (94.2)
Renin-angiotensin system inhibitors	4542 (69.1)	2803 (69.2)	3046 (68.6)	2985 (67.3)	4279 (70.3)
Calcium channel blocker	3637 (55.3)	2314 (57.1)	2571 (57.9)	2650 (59.7)	3665 (60.2)
B-blocker	4270 (65.0)	2584 (63.8)	2891 (65.1)	2868 (64.7)	3918 (64.3)
Diuretic therapy	4623 (70.3)	2663 (65.8)	2855 (64.3)	2735 (61.7)	3753 (61.6)
Thiazide & thiazide-like	972 (14.8)	675 (16.7)	778 (17.5)	653 (14.7)	839 (13.8)
Loop diuretic	4368 (66.4)	2460 (60.7)	2637 (59.4)	2532 (57.1)	3476 (57.1)
MRA	490 (7.5)	347 (8.6)	445 (10.0)	427 (9.6)	673 (11.0)
Amiloride	88 (1.3)	63 (1.6)	50 (1.1)	38 (0.9)	48 (0.8)
ARNI	0 (0.0)	0 (0.0)	2 (0.0)	11 (0.2)	106 (1.7)
SGLT2 inhibitors	0 (0.0)	1 (0.0)	6 (0.1)	13 (0.3)	137 (2.2)

Continuous variables are presented as mean (SD) or median [Q1-Q3], as appropriate. Categorical variables are presented as number (percentage).

ARNI: angiotensin receptor-neprilysin inhibitor; CKD: chronic kidney disease ; eGFR : estimated glomerular filtration rate ; MRA: mineralocorticoid receptor antagonist ; SGLT2 inhibitor: sodium-glucose cotransporter-2 inhibitor.

Table S3. Baseline characteristics, by calendar year, in patients with CKD stage 5-ND

CKD 5-ND	2011-2012 N = 3173	2013-2014 N = 2361	2015-2016 N = 2378	2017-2018 N = 2447	2019-2021 N = 3609
**Clinical and biological data**					
Age, years	72 [61, 80]	71 [61, 80]	72 [62, 81]	72 [61, 80]	73 [61, 80]
Gender, Women	1229 (38.7)	869 (36.8)	862 (36.2)	896 (36.6)	1293 (35.8)
Systolic blood pressure, mmHg	141 (22)	142 (22)	142 (21)	141 (21)	141 (20)
Diastolic blood pressure, mmHg	78 (12)	78 (13)	77 (12)	77 (12)	78 (12)
Body mass index, kg/m²	27.5 (5.6)	27.7 (5.5)	27.7 (6.0)	27.7 (5.8)	28.2 (6.3)
eGFR, mL/min per 1.73 m²	11 (3)	12 (2)	12 (2)	12 (2)	12 (2)
**Medical history**					
Hypertension	3085 (97.2)	2297 (97.3)	2301 (96.8)	2374 (97.0)	3516 (97.4)
Diabetes mellitus	1200 (37.8)	1012 (42.9)	1021 (42.9)	1027 (42.0)	1586 (43.9)
Any cardiovascular disease	1133 (35.7)	861 (36.5)	856 (36.0)	895 (36.6)	1304 (36.1)
Ischemic heart disease	851 (26.8)	655 (27.7)	607 (25.5)	630 (25.7)	943 (26.1)
Myocardial infarction	615 (19.4)	478 (20.2)	448 (18.8)	450 (18.4)	689 (19.1)
Cerebrovascular disease	477 (15.0)	400 (16.9)	423 (17.8)	433 (17.7)	619 (17.2)
Peripheral artery disease	373 (11.8)	299 (12.7)	285 (12.0)	289 (11.8)	382 (10.6)
Heart failure	721 (22.7)	585 (24.8)	621 (26.1)	627 (25.6)	1000 (27.7)
Atrial fibrillation / flutter	486 (15.3)	412 (17.5)	429 (18.0)	454 (18.6)	702 (19.5)
**Ongoing medications**					
Any antihypertensive therapy	3034 (95.6)	2258 (95.6)	2260 (95.0)	2330 (95.2)	3456 (95.8)
Renin-angiotensin system inhibitors	1970 (62.1)	1433 (60.7)	1441 (60.6)	1467 (60.0)	2268 (62.8)
Calcium channel blocker	2118 (66.8)	1599 (67.7)	1632 (68.6)	1678 (68.6)	2596 (71.9)
B-blocker	2143 (67.5)	1596 (67.6)	1646 (69.2)	1619 (66.2)	2492 (69.0)
Diuretic therapy	2334 (73.6)	1702 (72.1)	1681 (70.7)	1676 (68.5)	2519 (69.8)
Thiazide & thiazide like	296 (9.3)	233 (9.9)	237 (10.0)	202 (8.3)	284 (7.9)
Loop diuretic	2272 (71.6)	1646 (69.7)	1628 (68.5)	1618 (66.1)	2449 (67.9)
MRA	150 (4.7)	133 (5.6)	135 (5.7)	159 (6.5)	269 (7.5)
Amiloride	23 (0.7)	11 (0.5)	13 (0.5)	12 (0.5)	11(0.3)
ARNI	0 (0.0)	0 (0.0)	0 (0.0)	2 (0.1)	19 (0.5)
SGLT2i	0 (0.0)	0 (0.0)	0 (0.0)	3 (0.1)	26 (0.7)

Continuous variables are presented as mean (SD) or median [Q1-Q3], as appropriate. Categorical variables are presented as number (percentage).

ARNI: angiotensin receptor-neprilysin inhibitor; CKD: chronic kidney disease ; eGFR : estimated glomerular filtration rate ; MRA: mineralocorticoid receptor antagonist ; ND : non-dialysis ; SGLT2 inhibitor: sodium-glucose cotransporter-2 inhibitor.

Table S4. Baseline characteristics by calendar year, in patients on hemodialysis

HD	2011-2012<br>N = 3805	2013-2014<br>N = 1464	2015-2016<br>N = 1601	2017-2018<br>N = 1556	2019-2021<br>N = 2209
**Clinical and biological data**					
Age, years	68 [56, 77]	67 [55, 75]	68 [54, 76]	67 [55, 75]	69 [56, 77]
Gender, Women	1338 (35.2)	482 (32.9)	532 (33.2)	540 (34.7)	742 (33.6)
Systolic blood pressure, mmHg	140 (26)	146 (24)	146 (24)	146 (23)	145 (23)
Diastolic blood pressure, mmHg	73 (15)	76 (15)	76 (16)	76 (16)	76 (16)
Body mass index, kg/m²	25.9 (5.5)	26.5 (5.8)	26.7 (5.9)	26.4 (5.9)	26.7 (6.0)
**Medical history**					
Hypertension	3609 (94.8)	1414 (96.6)	1548 (96.7)	1509 (97.0)	2176 (98.5)
Diabetes mellitus	1626 (42.7)	659 (45.0)	734 (45.8)	690 (44.3)	1071 (48.5)
Any cardiovascular disease	1770 (46.5)	615 (42.0)	642 (40.1)	604 (38.8)	879 (39.8)
Ischemic heart disease	1327 (34.9)	461 (31.5)	456 (28.5)	416 (26.7)	644 (29.2)
Myocardial infarction	985 (25.9)	341 (23.3)	331 (20.7)	309 (19.9)	471 (21.3)
Cerebrovascular disease	705 (18.5)	275 (18.8)	276 (17.2)	268 (17.2)	387 (17.5)
Peripheral artery disease	874 (23.0)	267 (18.2)	261 (16.3)	268 (17.2)	336 (15.2)
Heart failure	1197 (31.5)	461 (31.5)	521 (32.5)	498 (32.0)	821 (37.2)
Atrial fibrillation / flutter	922 (24.2)	298 (20.4)	366 (22.9)	331 (21.3)	533 (24.1)
**Ongoing medications**					
Any antihypertensive therapy	3315 (87.1)	1380 (94.3)	1502 (93.8)	1473 (94.7)	2141 (96.9)
Renin-angiotensin system inhibitors	1695 (44.5)	683 (46.7)	773 (48.3)	688 (44.2)	1142 (51.7)
Calcium channel blocker	1789 (47.0)	970 (66.3)	1049 (65.5)	1084 (69.7)	1562 (70.7)
B-blocker	2511 (66.0)	1089 (74.4)	1202 (75.1)	1159 (74.5)	1703 (77.1)
Diuretic therapy	2274 (59.8)	1138 (77.7)	1271 (79.4)	1244 (79.9)	1857 (84.1)
Thiazide & thiazide-like	81 (2.1)	62 (4.2)	100 (6.2)	73 (4.7)	151 (6.8)
Loop diuretic	1358 (35.7)	927 (63.3)	1068 (66.7)	1035 (66.5)	1510 (68.4)
MRA	62 (1.6)	54 (3.7)	68 (4.2)	64 (4.1)	145 (6.6)
Amiloride	3 (0.1)	1 (0.1)	2 (0.1)	0 (0.0)	2 (0.1)
ARNI	0 (0.0)	0 (0.0)	0 (0.0)	0 (0.0)	13 (0.6)
SGLT2 inhibitors	0 (0.0)	0 (0.0)	0 (0.0)	0 (0.0)	10 (0.5)

Continuous variables are presented as mean (SD) or median [Q1-Q3], as appropriate. Categorical variables are presented as number (percentage).

ARNI: angiotensin receptor-neprilysin inhibitor; CKD: chronic kidney disease ; eGFR : estimated glomerular filtration rate ; MRA: mineralocorticoid receptor antagonist ; SGLT2 inhibitor: sodium-glucose cotransporter-2 inhibitor.

Table S5. Baseline characteristics by calendar year, in patients on peritoneal dialysis

PD	2011-2012 N = 1149	2013-2014 N = 719	2015-2016 N = 746	2017-2018 N = 727	2019-2021 N = 1170
**Clinical and biological data**					
Age, years	67 [55, 76]	66 [53, 74]	67 [52, 75]	68 [55, 76]	68 [53, 77]
Gender, Women	382 (33.2)	237 (33.0)	252 (33.8)	224 (30.8)	345 (29.5)
Systolic blood pressure, mmHg	137 (22)	137 (21)	139 (21)	136 (20)	136 (20)
Diastolic blood pressure, mmHg	78 (12)	78 (12)	79 (12)	78 (13)	79 (12)
Body mass index, kg/m²	26.3 (4.9)	26.3 (4.3)	25.9 (4.6)	26.0 (5.1)	26.4 (4.7)
**Medical history**					
Hypertension	1135 (98.8)	713 (99.2)	739 (99.1)	719 (98.9)	1160 (99.1)
Diabetes mellitus	457 (39.8)	274 (38.1)	276 (37.0)	313 (43.1)	466 (39.8)
Any cardiovascular disease	472 (41.1)	259 (36.0)	241 (32.3)	254 (34.9)	372 (31.8)
Ischemic heart disease	380 (33.1)	197 (27.4)	183 (24.5)	193 (26.5)	267 (22.8)
Myocardial infarction	280 (24.4)	144 (20.0)	125 (16.8)	139 (19.1)	199 (17.0)
Cerebrovascular disease	194 (16.9)	111 (15.4)	115 (15.4)	118 (16.2)	180 (15.4)
Peripheral artery disease	181 (15.8)	101 (14.0)	84 (11.3)	93 (12.8)	121 (10.3)
Heart failure	292 (25.4)	179 (24.9)	181 (24.3)	186 (25.6)	300 (25.6)
Atrial fibrillation / flutter	203 (17.7)	113 (15.7)	128 (17.2)	153 (21.0)	214 (18.3)
**Ongoing medications**					
Any antihypertensive therapy	1103 (96.0)	701 (97.5)	730 (97.9)	708 (97.4)	1148 (98.1)
Renin-angiotensin system inhibitors	634 (55.2)	382 (53.1)	373 (50.0)	357 (49.1)	605 (51.7)
Calcium channel blocker	693 (60.3)	507 (70.5)	554 (74.3)	538 (74.0)	871 (74.4)
B-blocker	839 (73.0)	540 (75.1)	565 (75.7)	551 (75.8)	882 (75.4)
Diuretic therapy	921 (80.2)	621 (86.4)	655 (87.8)	632 (86.9)	1036 (88.5)
Thiazide & thiazide-like	41 (3.6)	41 (5.7)	62 (8.3)	52 (7.2)	105 (9.0)
Loop diuretic	653 (56.8)	543 (75.5)	577 (77.3)	589 (81.0)	929 (79.4)
MRA	43 (3.7)	25 (3.5)	28 (3.8)	43 (5.9)	78 (6.7)
Amiloride	2 (0.2)	1 (0.1)	3 (0.4)	1 (0.1)	2 (0.2)
ARNI	0 (0.0)	0 (0.0)	0 (0.0)	2 (0.3)	4 (0.3)
SGLT2 inhibitors	0 (0.0)	0 (0.0)	0 (0.0)	0 (0.0)	3 (0.3)

Continuous variables are presented as mean (SD) or median [Q1-Q3], as appropriate. Categorical variables are presented as number (percentage).

ARNI: angiotensin receptor-neprilysin inhibitor; CKD: chronic kidney disease ; eGFR : estimated glomerular filtration rate ; MRA: mineralocorticoid receptor antagonist ; SGLT2 inhibitor: sodium-glucose cotransporter-2 inhibitor.

Table S6. Number of events, incidence rates per 100-PY, age-sex adjusted incidence rate ratios of heart failure-related hospitalization, by calendar year.

HF hospitalization	N events / N individuals	IR per 100-PY (95%CI)	% change*	Adjusted IRR ** (95%CI)	P for trend	Std. IRR*** (95%CI)
**CKD stage 4**						
2011-2012	1273 / 6574	5.99 (5.67-6.33)	Ref	Ref	<0.01	4.45 (4.20-4.71)
2013-2014	841 / 4050	6.47 (6.04-6.91)	8.0	1.05 (0.96-1.14)		5.16 (4.79-5.54)
2015-2016	863 / 4440	6.76 (6.32-7.22)	12.9	1.06 (0.97-1.15)		5.41 (5.03-5.81)
2017-2018	830 / 4436	7.67 (7.16-8.21)	28.0	1.15 (1.06-1.26)		6.52 (6.05-7.03)
2019-2021	627 / 6091	9.04 (8.35-9.77)	50.9	1.31 (1.20-1.45)		9.41 (8.61-10.28)
**CKD stage 5**						
2011-2012	470 / 3173	8.37 (7.64-9.15)	Ref	Ref	<0.01	6.22 (5.67-6.81)
2013-2014	380 / 2361	9.52 (8.60-10.51)	13.7	1.14 (0.99-1.30)		7.59 (6.83-8.40)
2015-2016	367 / 2378	9.39 (8.46-10.38)	12.2	1.07 (0.94-1.23)		7.52 (6.75-8.35)
2017-2018	371 / 2447	9.85 (8.88-10.89)	17.7	1.16 (1.01-1.32)		8.38 (7.52-9.30)
2019-2021	350 / 3609	11.37 (10.22-12.61)	35.8	1.32 (1.15-1.52)		11.84 (10.56-13.24)
**Hemodialysis**						
2011-2012	580 / 3805	4.90 (4.51-5.31)	Ref	Ref	<0.01	3.64 (3.35-3.95)
2013-2014	214 / 1464	5.06 (4.41-5.77)	3.3	1.08 (0.92-1.26)		4.03 (3.51-4.61)
2015-2016	215 / 1601	5.04 (4.39-5.74)	2.9	1.05 (0.89-1.22)		4.04 (3.51-4.61)
2017-2018	194 / 1556	5.68 (4.92-6.52)	15.9	1.18 (1.00-1.39)		4.83 (4.17-5.56)
2019-2021	146 / 2209	6.37 (5.39-7.46)	30.0	1.26 (1.05-1.51)		6.64 (5.59- 7.82)
**Peritoneal dialysis**						
2011-2012	106 / 1149	5.23 (4.29-6.29)	Ref	Ref	<0.01	3.88 (3.19-4.68)
2013-2014	65 / 719	5.28 (4.10-6.67)	1.0	1.00 (0.79-1.24)		4.21 (3.26-5.32)
2015-2016	68 / 746	5.39 (4.21-6.78)	3.1	0.74 (0.58-0.94)		4.32 (3.37-5.44)
2017-2018	63 / 727	6.27 (4.84-7.95)	19.9	0.66 (0.51-0.86)		5.33 (4.11-6.77)
2019-2021	47 / 1170	5.28 (3.91-6.94)	1.0	0.71 (0.54-0.93)		5.50 (4.06- 7.25)

*Percentage risk reduction compared to 2011-2012. A negative value indicates a decrease in rate, whereas a positive value indicates an increase in rate of heart failure.

**Incidence rate ratios were estimated using Poisson regression, adjusted for age and sex.

*** Standardized incidence rate ratio compared to a 1:10 age- and sex-matched general population. Standardized incidence rate ratios were calculated according to indirect standardization by taking the observed number of events divided by the expected number of events for CKD populations based on the observed rates in the age-, sex- matched general population, with 95% confidence intervals computed assuming a Poisson distribution.

CI: confidence intervals ; IR: incidence rate ; Std IRR: standardized incidence rate ratio.

Table S7. Number of events, incidence rates per 100-PY, age-sex adjusted incidence rate ratios of death due to heart failure, by calendar year.

HF death	N events / N individuals	IR per 100-PY (95%CI)	% change*	Adjusted IRR** (95%CI)	P for trend	Std. IRR*** (95%CI)
**CKD stage 4**						
2011-2012	600 / 6574	2.62 (2.42-2.84)	Ref	Ref	<0.01	10.26 (9.38-11.19)
2013-2014	392 / 4050	2.77 (2.50-3.05)	5.7	1.02 (0.89-1.15)		11.97 (10.67-13.40)
2015-2016	367 / 4440	2.65 (2.38-2.93)	1.1	0.94 (0.82-1.06)		12.69 (11.23-14.31)
2017-2018	306 / 4436	2.60 (2.32-2.90)	-0.8	0.86 (0.75-0.99)		14.03 (12.21-16.08)
2019-2021	151 / 6091	2.62 (2.42-2.84)	-21.0	0.66 (0.55-0.79)		19.41 (15.83-23.73)
**CKD stage 5**						
2011-2012	226 / 3173	3.79 (3.32-4.30)	Ref	Ref	0.08	14.81 (12.89-16.91)
2013-2014	172 / 2361	4.04 (3.46-4.67)	6.6	1.06 (0.87-1.29)		17.48 (14.85-20.44)
2015-2016	154 / 2378	3.66 (3.11-4.27)	-3.4	0.88 (0.72-1.08)		17.56 (14.75-20.75)
2017-2018	150 / 2447	3.73 (3.16-4.36)	-1.6	0.94 (0.76-1.15)		20.15 (16.79-24.02)
2019-2021	109 / 3609	3.38 (2.79-4.06)	-10.8	0.84 (0.67-1.05)		31.75 (25.25-39.65)
**Hemodialysis**						
2011-2012	376 / 3805	2.93 (2.64-3.23)	Ref	Ref	<0.01	11.45 (10.26-12.73)
2013-2014	130 / 1464	2.80 (2.35-3.31)	-4.4	1.02 (0.83-1.24)		12.13 (10.08-14.47)
2015-2016	93 / 1601	2.01 (1.63-2.44)	-31.4	0.70 (0.56-0.88)		9.62 (7.72-11.84)
2017-2018	70 / 1556	1.92 (1.51-2.41)	-34.5	0.67 (0.52-0.86)		10.38 (8.04-13.19)
2019-2021	48 / 2209	2.01 (1.49-2.63)	-31.4	0.66 (0.48-0.88)		18.83 (13.66-25.39)
**Peritoneal dialysis**						
2011-2012	76 / 1149	3.59 (2.84-4.46)	Ref	Ref	<0.01	14.03 (11.07-17.48)
2013-2014	22 / 719	1.70 (1.08-2.51)	-52.6	0.48 (0.29-0.75)		7.36 (14.68-10.93)
2015-2016	36 / 746	2.70 (1.91-3.68)	-24.8	0.73 (0.48-1.07)		12.96 (9.12-17.79)
2017-2018	27 / 727	2.59 (1.73-3.69)	-27.9	0.62 (0.39-0.95)		13.98 (9.28-20.13)
2019-2021	11 / 1170	1.21 (0.63-2.07)	-66.3	0.31 (0.15-0.55)		11.36 (5.84-19.76)

*Percentage risk reduction compared to 2011-2012. A negative value indicates a decrease in rate, whereas a positive value indicates an increase in rate of heart failure death.

** Incidence rate ratios were estimated using Poisson regression, adjusted for age and sex.

*** Standardized incidence rate ratio compared to a 1:10 age- and sex-matched general population. Standardized incidence rate ratios were calculated according to indirect standardization by taking the observed number of events divided by the expected number of events for CKD populations based on the observed rates in the age-, sex- matched general population, with 95% confidence intervals computed assuming a Poisson distribution.

CI: confidence intervals ; IR: incidence rate ; Std IRR: standardized incidence rate ratio.

Table S8. Number of events, incidence rates per 100-PY, age-sex adjusted incidence rate ratios of all-cause mortality, by calendar year.

All-cause mortality	N events / N individuals	IR per 100-PY (95%CI)	% change*	Adjusted IRR** (95%CI)	P for trend	Std. IRR*** (95%CI)
**CKD stage 4**						
2011-2012	2312 / 6574	10.11 (9.70-10.53)	0.0	Ref	<0.01	2.05 (1.97-2.14)
2013-2014	1417 / 4050	10.00 (9.48-10.53)	-1.1	0.96 (0.90-1.02)		2.14 (2.03-2.26)
2015-2016	1412 / 4440	10.18 (9.66-10.72)	0.7	0.94 (0.88-1.01)		2.15 (2.04-2.27)
2017-2018	1204 / 4436	10.21 (9.65-10.80)	1.0	0.90 (0.84-0.97)		2.23 (2.10-2.36)
2019-2021	688 / 6091	9.42 (8.74-10.14)	-6.8	0.81 (0.75-0.88)		2.18 (2.02-2.36)
**CKD stage 5**						
2011-2012	1037 / 3173	17.38 (16.34-18.46)	0.0	Ref	0.06	3.53 (3.31-3.75)
2013-2014	752 / 2361	17.66 (16.42-18.95)	1.6	1.01 (0.92-1.11)		3.78 (3.51-4.06)
2015-2016	716 / 2378	17.02 (15.81-18.30)	-2.1	0.91 (0.83-1.00)		3.60 (3.34-3.87)
2017-2018	691 / 2447	17.18 (15.93-18.49)	-1.2	0.95 (0.86-1.05)		3.74 (3.47-4.04)
2019-2021	540 / 3609	16.75 (15.38-18.21)	-3.6	0.92 (0.83-1.03)		3.88 (3.55-4.23)
**Hemodialysis**						
2011-2012	2618 / 3805	20.39 (19.61-21.18)	0.0	Ref	<0.01	4.14 (3.98-4.30)
2013-2014	800 / 1464	17.25 (16.08-18.47)	-15.4	0.89 (0.82-0.96)		3.69 (3.44-3.96)
2015-2016	753 / 1601	16.25 (15.11-17.43)	-20.3	0.81 (0.75-0.88)		3.43 (3.19-3.69)
2017-2018	573 / 1556	15.73 (14.48-17.05)	-22.9	0.79 (0.72-0.86)		3.43 (3.15-3.72)
2019-2021	347 / 2209	14.50 (13.03-16.08)	-28.9	0.69 (0.62-0.77)		3.36 (3.01-3.73)
**Peritoneal dialysis**						
2011-2012	443 / 1149	20.91 (19.02-22.92)	0.0	Ref	<0.01	4.24 (3.86-4.65)
2013-2014	221 / 719	17.09 (14.93-19.44)	-18.3	0.82 (0.70-0.96)		3.66 (3.19-4.16)
2015-2016	233 / 746	17.50 (15.34-19.84)	-16.3	0.80 (0.69-0.94)		3.70 (3.24-4.20)
2017-2018	194 / 727	18.59 (16.10-21.33)	-11.1	0.77 (0.65-0.91)		4.05 (3.51-4.66)
2019-2021	135 / 1170	14.86 (12.49-17.51)	-28.9	0.65 (0.53-0.78)		3.44 (2.89-4.06)

*Percentage risk reduction compared to 2011-2012. A negative value indicates a decrease in rate, whereas a positive value indicates an increase in rate of heart failure death.

** Incidence rate ratios were estimated using Poisson regression, adjusted for age and sex.

*** Standardized incidence rate ratio compared to a 1:10 age- and sex-matched general population. Standardized incidence rate ratios were calculated according to indirect standardization by taking the observed number of events divided by the expected number of events for CKD populations based on the observed rates in the age-, sex- matched general population, with 95% confidence intervals computed assuming a Poisson distribution.

CI: confidence intervals ; IR: incidence rate ; Std IRR: standardized incidence rate ratio.

Table S9. Sensitivity analyses (exclusion of non-matched CKD patients): incidence rates and standardized incidence rate ratios of heart failure-related hospitalization.

HF hospitalization	N events / N individuals	IR per 100-PY (95%CI)	% change*	Std. IRR** (95%CI)
**CKD stage 4**				
2011-2012	1256 / 5819	6.74 (6.37-7.12)	Ref	5.00 (4.72-5.30)
2013-2014	825 / 3530	7.26 (6.77-7.76)	7.7	5.79 (5.38-6.22)
2015-2016	844 / 3798	7.69 (7.18-8.22)	14.1	6.16 (5.72-6.61)
2017-2018	802 / 3752	8.77 (8.18-9.39)	30.1	7.46 (6.90- 8.04)
2019-2021	600 / 5022	10.45 (9.64-11.31)	54.3	10.89 (9.94-11.91)
**CKD stage 5**				
2011-2012	460 / 2739	9.16 (8.35-10.02)	Ref	6.80 (6.19-7.46)
2013-2014	372 / 1958	10.78 (9.72-11.91)	17.6	8.60 (7.73-9.53)
2015-2016	359 / 1962	10.65 (9.59-11.79)	16.5	8.53 (7.66-9.48)
2017-2018	347 / 1937	11.12 (9.99-12.34)	22.0	9.46 (8.46-10.54)
2019-2021	330 / 2751	13.70 (12.28-15.23)	50.5	14.27 (12.69-16.00)
**Hemodialysis**				
2011-2012	540 / 3106	5.67 (5.20-6.16)	Ref	4.21 (3.86-4.58)
2013-2014	193 / 1109	6.17 (5.34-7.08)	8.8	4.92 (4.25-5.66)
2015-2016	196 / 1155	6.30 (5.46-7.22)	11.1	5.04 (4.36-5.80)
2017-2018	171 / 1065	7.35 (6.30-8.50)	29.6	6.24 (5.34- 7.25)
2019-2021	115 / 1490	7.22 (5.98-8.62)	27.3	7.52 (6.20- 9.02)
**Peritoneal dialysis**				
2011-2012	96 / 915	6.30 (5.12-7.65)	Ref	4.68 (3.81-5.69)
2013-2014	58 / 526	6.24 (4.76-7.98)	-1.0	4.97 (3.80-6.37)
2015-2016	58 / 505	6.55 (5.01-8.39)	4.0	5.25 (4.01-6.73)
2017-2018	56 / 503	7.63 (5.80-9.81)	21.1	6.49 (4.92- 8.35)
2019-2021	39 / 732	6.64 (4.77-8.95)	5.4	6.92 (4.96- 9.35)

*Percentage risk reduction compared to 2011-2012. A negative value indicates a decrease in rate, whereas a positive value indicates an increase in rate of heart failure.
** Standardized incidence rate ratio compared to a 1:10 age- and sex-matched general population. Standardized incidence rate ratios were calculated according to indirect standardization by taking the observed number of events divided by the expected number of events for CKD populations based on the observed rates in the age-, sex- matched general population, with 95% confidence intervals computed assuming a Poisson distribution.
CI: confidence intervals ; IR: incidence rate ; Std IRR: standardized incidence rate ratio.

Table S10. Sensitivity analyses (exclusion of non-matched CKD patients): incidence rates and standardized incidence rate ratios of death due to heart failure.

HF death	N events / N individuals	IR per 100-PY (95%CI)	% change*	Std. IRR** (95%CI)
**CKD stage 4**				
2011-2012	597 / 5819	2.95 (2.72-3.19)	Ref	11.54 (10.55-12.59)
2013-2014	390 / 3530	3.12 (2.82-3.44)	5.8	13.48 (12.02-15.10)
2015-2016	359 / 3798	2.98 (2.68-3.30)	1.0	14.27 (12.62-16.11)
2017-2018	304 / 3752	3.02 (2.69-3.37)	2.4	16.30 (14.19-18.69)
2019-2021	147 / 5022	2.42 (2.04-2.83)	-18.0	22.67 (18.45-27.76)
**CKD stage 5**				
2011-2012	226 / 2739	4.21 (3.68-4.78)	Ref	16.45 (14.32-18.78)
2013-2014	171 / 1958	4.60 (3.95-5.33)	9.3	19.92 (16.92-23.30)
2015-2016	153 / 1962	4.19 (3.56-4.88)	-0.5	20.08 (16.86-23.74)
2017-2018	150 / 1937	4.46 (3.79-5.21)	5.9	24.10 (20.08-28.73)
2019-2021	108 / 2751	4.24 (3.49-5.09)	0.7	39.84 (31.66-49.78)
**Hemodialysis**				
2011-2012	358 / 3106	3.43 (3.08-3.79)	Ref	13.39 (11.98-14.93)
2013-2014	119 / 1109	3.43 (2.85-4.08)	0.0	14.83 (12.22-17.81)
2015-2016	84 / 1155	2.45 (1.97-3.02)	-28.6	11.77 (9.34-14.61)
2017-2018	63 / 1065	2.50 (1.94-3.17)	-27.1	13.52 (10.33-17.38)
2019-2021	40 / 1490	2.39 (1.73-3.21)	-30.3	22.47 (15.86-30.99)
**Peritoneal dialysis**				
2011-2012	73 / 915	4.56 (3.59-5.69)	Ref	17.84 (14.01-22.32)
2013-2014	22 / 526	2.23 (1.43-3.30)	-51.1	9.67 (6.15-14.35)
2015-2016	34 / 505	3.60 (2.52-4.95)	-21.1	17.25 (12.02-23.88)
2017-2018	26 / 503	3.40 (2.26-4.88)	-25.4	18.39 (12.11-26.63)
2019-2021	10 / 732	1.66 (0.83-2.91)	-63.6	15.57 (7.72-27.72)

*Percentage risk reduction compared to 2011-2012. A negative value indicates a decrease in rate, whereas a positive value indicates an increase in rate of heart failure death.
** Standardized incidence rate ratio compared to a 1:10 age- and sex-matched general population. Standardized incidence rate ratios were calculated according to indirect standardization by taking the observed number of events divided by the expected number of events for CKD populations based on the observed rates in the age-, sex- matched general population, with 95% confidence intervals computed assuming a Poisson distribution.
CI: confidence intervals ; IR: incidence rate ; Std IRR: standardized incidence rate ratio.

Table S11. Sensitivity analyses: standardized incidence rate ratios of HF-related hospitalization and death due to HF, after re-matching CKD patients with another set of controls from the general population

	HF hospitalization Std. IRR (95%CI)*	Death due to HF Std. IRR (95%CI)*
**CKD stage 4**		
2011-2012	4.50 (4.25-4.76)	10.15 (9.32-11.03)
2013-2014	5.17 (4.82-5.54)	11.90 (10.68-13.22)
2015-2016	5.47 (5.10-5.86)	12.52 (11.18-13.99)
2017-2018	6.46 (6.01-6.93)	14.42 (12.69-16.32)
2019-2021	9.81 (9.02-10.66)	19.66 (16.33-23.52)
**CKD stage 5**		
2011-2012	6.28 (5.73-6.88)	14.65 (12.79-16.68)
2013-2014	7.61 (6.86-8.41)	17.38 (14.84-20.22)
2015-2016	7.60 (6.83-8.41)	17.33 (14.64-20.34)
2017-2018	8.29 (7.46-9.19)	20.71 (17.41-24.46)
2019-2021	12.35 (11.05-13.74)	32.14 (25.99-39.38)
**Hemodialysis**		
2011-2012	3.68 (3.39-3.99)	11.32 (10.18-12.55)
2013-2014	4.04 (3.52-4.62)	12.06 (10.06-14.32)
2015-2016	4.08 (3.55-4.65)	9.49 (7.66-11.62)
2017-2018	4.78 (4.13-5.50)	10.67 (8.31-13.47)
2019-2021	6.92 (5.84- 8.13)	19.06 (13.99-25.35)
**Peritoneal dialysis**		
2011-2012	3.93 (3.22-4.72)	13.88 (10.97-17.26)
2013-2014	4.22 (3.27-5.33)	7.32 (4.66-10.85)
2015-2016	4.36 (3.40-5.49)	12.79 (9.03-17.50)
2017-2018	5.28 (4.08-6.70)	14.38 (9.58-20.60)
2019-2021	5.73 (4.24- 7.54)	11.50 (5.94-19.85)

* Standardized incidence rate ratio compared to a 1:10 age- and sex-matched general population. Standardized incidence rate ratios were calculated according to indirect standardization by taking the observed number of events divided by the expected number of events for CKD populations based on the observed rates in the age-, sex- matched general population, with 95% confidence intervals computed assuming a Poisson distribution.
CI: confidence intervals ; Std IRR: standardized incidence rate ratio.

Table S12. Sensitivity analyses: standardized incidence rate ratios of heart failure-related hospitalization and, in patients without history of heart failure

HF hospitalization	N events / N individuals	IR per 100-PY (95%CI)	% change*	Std. IRR** (95%CI)
**CKD stage 4**				
2011-2012	551 / 4968	3.06 (2.81-3.32)	0.0	3.07 (2.81-3.34)
2013-2014	320 / 2990	2.95 (2.63-3.28)	-3.6	3.30 (2.94-3.69)
2015-2016	326 / 3245	3.16 (2.83-3.52)	3.3	3.86 (3.44-4.31)
2017-2018	253 / 3197	2.95 (2.60-3.33)	-3.6	4.28 (3.75-4.87)
2019-2021	172 / 4309	3.28 (2.82-3.80)	7.2	6.81 (5.78-7.98)
**CKD stage 5**				
2011-2012	192 / 2452	4.02 (3.48-4.62)	0.0	4.04 (3.49-4.64)
2013-2014	157 / 1776	4.81 (4.10-5.60)	19.7	5.38 (4.57-6.29)
2015-2016	154 / 1757	4.83 (4.10-5.63)	20.1	5.89 (4.99-6.89)
2017-2018	154 / 1820	5.10 (4.34-5.95)	26.9	7.42 (6.27-8.70)
2019-2021	119 / 2609	5.08 (4.22-6.05)	26.4	10.53 (8.67-12.68)
**Hemodialysis**				
2011-2012	263 / 2608	3.00 (2.65-3.38)	0.0	3.01 (2.66-3.39)
2013-2014	93 / 1003	2.96 (2.40-3.61)	-1.3	3.32 (2.68-4.05)
2015-2016	91 / 1080	2.98 (2.41-3.64)	-0.7	3.64 (2.93-4.45)
2017-2018	68 / 1058	2.86 (2.23-3.59)	-4.7	4.15 (3.23-5.24)
2019-2021	47 / 1388	3.15 (2.33-4.14)	5.0	6.54 (4.81- 8.65)
**Peritoneal dialysis**				
2011-2012	48 / 857	2.95 (2.19-3.87)	0.0	2.96 (2.20-3.88)
2013-2014	25 / 540	2.56 (1.68-3.69)	-13.2	2.86 (1.88-4.14)
2015-2016	24 / 565	2.39 (1.55-3.47)	-19.0	2.91 (1.90-4.24)
2017-2018	29 / 541	3.68 (2.50-5.19)	24.7	5.35 (3.62-7.56)
2019-2021	16 / 870	2.37 (1.39-3.72)	-19.7	4.91 (2.87-7.75)

*Percentage risk reduction compared to 2011-2012. A negative value indicates a decrease in rate, whereas a positive value indicates an increase in rate of heart failure death.
** Standardized incidence rate ratio compared to a 1:10 age- and sex-matched general population. Standardized incidence rate ratios were calculated according to indirect standardization by taking the observed number of events divided by the expected number of events for CKD populations based on the observed rates in the age-, sex- matched general population, with 95% confidence intervals computed assuming a Poisson distribution.
CI: confidence intervals ; IR: incidence rate ; Std IRR: standardized incidence rate ratio.

Table S13. Sensitivity analyses: standardized incidence rate ratios of death due to heart failure, in patients without history of heart failure

HF death	N events / N individuals	IR per 100-PY (95%CI)	% change*	Std. IRR** (95%CI)
**CKD stage 4**				
2011-2012	250 / 4968	1.33 (1.18-1.51)	0.0	6.90 (6.04- 7.85)
2013-2014	139 / 2990	1.24 (1.04-1.45)	-6.8	7.63 (6.35- 9.10)
2015-2016	142 / 3245	1.33 (1.13-1.57)	0.0	9.24 (7.67-11.05)
2017-2018	84 / 3197	0.95 (0.76-1.17)	-28.6	9.72 (7.59-12.32)
2019-2021	36 / 4309	0.67 (0.48-0.92)	-49.6	13.47 (9.12-19.43)
**CKD stage 5**				
2011-2012	113 / 2452	2.29 (1.90-2.74)	0.0	11.86 (9.76-14.26)
2013-2014	75 / 1776	2.22 (1.76-2.77)	-3.1	13.71 (10.74-17.24)
2015-2016	52 / 1757	1.58 (1.19-2.05)	-31.0	10.94 (8.14-14.36)
2017-2018	48 / 1820	1.54 (1.14-2.02)	-32.8	15.72 (11.45-21.09)
2019-2021	38 / 2609	1.59 (1.14-2.15)	-30.6	31.83 (21.73-45.61)
**Hemodialysis**				
2011-2012	188 / 2608	2.04 (1.77-2.35)	0.0	10.57 (9.08-12.24)
2013-2014	61 / 1003	1.84 (1.42-2.35)	-9.8	11.38 (8.69-14.63)
2015-2016	44 / 1080	1.38 (1.01-1.82)	-32.4	9.53 (6.92-12.78)
2017-2018	28 / 1058	1.13 (0.76-1.60)	-44.6	11.53 (7.65-16.66)
2019-2021	15 / 1388	0.99 (0.57-1.57)	-51.5	19.71 (11.03-32.73)
**Peritoneal dialysis**				
2011-2012	39 / 857	2.34 (1.68-3.15)	0.0	12.10 (8.66-16.36)
2013-2014	9 / 540	0.90 (0.43-1.62)	-61.5	5.54 (2.65-10.03)
2015-2016	15 / 565	1.46 (0.84-2.33)	-37.6	10.12 (5.79-16.26)
2017-2018	7 / 541	0.87 (0.38-1.69)	-62.8	8.91 (3.80-17.42)
2019-2021	2 / 870	0.29 (0.05-0.90)	-87.6	5.85 (0.96-18.40)

*Percentage risk reduction compared to 2011-2012. A negative value indicates a decrease in rate, whereas a positive value indicates an increase in rate of heart failure death.
** Standardized incidence rate ratio compared to a 1:10 age- and sex-matched general population. Standardized incidence rate ratios were calculated according to indirect standardization by taking the observed number of events divided by the expected number of events for CKD populations based on the observed rates in the age-, sex- matched general population, with 95% confidence intervals computed assuming a Poisson distribution.
CI: confidence intervals ; IR: incidence rate ; Std IRR: standardized incidence rate ratio.

Table S14. Number of events, incidence rates and age adjusted incidence rate ratios of heart failure-related hospitalization, by CKD cohort, calendar year and sex.

	Men			Women		
	N events	IR per 100-PY (95%CI)	Std. IRR (95%CI)	N events	IR per 100-PY (95%CI)	Std. IRR (95%CI)
**CKD stage 4**						
2011-2012	834/4218	6.44 (6.02-6.89)	4.23 (3.94-4.54)	439/2356	5.29 (4.81-5.80)	5.04 (4.56-5.55)
2013-2014	561/2531	7.46 (6.86-8.10)	5.27 (4.82-5.75)	280/1519	5.10 (4.53-5.72)	5.20 (4.58- 5.89)
2015-2016	544/2773	7.19 (6.60-7.81)	5.08 (4.64-5.55)	319/1667	6.13 (5.49-6.83)	6.34 (5.61- 7.14)
2017-2018	548/2851	8.15 (7.49-8.86)	6.04 (5.50-6.61)	282/1585	6.88 (6.11-7.72)	7.93 (6.94- 9.04)
2019-2021	416/3883	9.57 (8.68-10.52)	9.01 (8.08-10.03)	211/2208	8.15 (7.10-9.30)	10.50 (8.97-12.25)
**CKD stage 5-ND**						
2011-2012	297/1944	9.71 (8.65-10.86)	6.38 (5.67-7.14)	173/1229	6.77 (5.81-7.83)	6.45 (5.52-7.49)
2013-2014	223/1492	9.46 (8.27-10.75)	6.68 (5.82-7.62)	157/869	9.62 (8.19-11.20)	9.81 (8.30-11.51)
2015-2016	232/1516	9.96 (8.73-11.30)	7.04 (6.15-8.01)	135/862	8.55 (7.18-10.07)	8.83 (7.38-10.49)
2017-2018	220/1551	9.87 (8.62-11.23)	7.31 (6.35-8.36)	151/896	9.83 (8.35-11.48)	11.33 (9.51-13.40)
2019-2021	211/2316	11.17 (9.73-12.75)	10.52 (9.09-12.12)	139/1293	11.69 (9.85-13.74)	15.06 (12.49-18.03)
**Hemodialysis**						
2011-2012	393/2467	5.22 (4.72-5.76)	3.43 (3.10-3.79)	187/1338	4.34 (3.75-4.99)	4.14 (3.56-4.77)
2013-2014	148/982	5.38 (4.56-6.30)	3.80 (3.21-4.46)	66/482	4.46 (3.47-5.62)	4.55 (3.52- 5.76)
2015-2016	154/1069	5.45 (4.64-6.36)	3.85 (3.27-4.51)	61/532	4.23 (3.25-5.38)	4.37 (3.35- 5.59)
2017-2018	124/1016	5.67 (4.73-6.72)	4.20 (3.49-5.00)	70/540	5.71 (4.47-7.15)	6.58 (5.12- 8.32)
2019-2021	101/1467	6.77 (5.54-8.18)	6.38 (5.19- 7.76)	45/742	5.62 (4.14-7.43)	7.25 (5.28- 9.68)
**Peritoneal dialysis**						
2011-2012	75/767	5.67 (4.48-7.05)	3.73 (2.94-4.64)	31/382	4.40 (3.03-6.13)	4.19 (2.88-5.85)
2013-2014	44/482	5.16 (3.78-6.84)	3.65 (2.67-4.84)	21/237	5.54 (3.49-8.26)	5.65 (3.56- 8.45)
2015-2016	44/494	5.16 (3.78-6.83)	3.64 (2.67-4.84)	24/252	5.88 (3.83-8.56)	6.08 (3.95- 8.88)
2017-2018	46/503	6.37 (4.70-8.39)	4.72 (3.47-6.23)	17/224	6.01 (3.58-9.33)	6.92 (4.12-10.81)
2019-2021	32/825	5.12 (3.54-7.10)	4.82 (3.33- 6.72)	15/345	5.66 (3.26-9.03)	7.29 (4.18-11.71)

Standardized incidence rate ratio compared to a 1:10 age- and sex-matched general population. Standardized incidence rate ratios were calculated according to indirect standardization by taking the observed number of events divided by the expected number of events for CKD populations based on the observed rates in the age-, sex- matched general population, with 95% confidence intervals computed assuming a Poisson distribution.
CI: confidence intervals ; IR: incidence rate ; Std IRR: standardized incidence rate ratio.

Table S15. Number of events, incidence rates and age adjusted incidence rate ratios of heart failure-related hospitalization, by CKD cohort, calendar year and diuretic use.

	No diuretic use			Diuretic use		
	**N events**	**IR per 100-PY (95%CI)**	**Std. IRR (95%CI)**	**N events**	**IR per 100-PY (95%CI)**	**Std. IRR (95%CI)**
**CKD stage 4**						
2011-2012	179/1951	2.40 (2.06-2.77)	2.75 (2.36-3.18)	1094/4623	7.93 (7.47-8.41)	2.12 (1.99-2.26)
2013-2014	118/1387	2.28 (1.90-2.72)	2.83 (2.34-3.38)	723/2663	9.22 (8.57-9.91)	2.54 (2.34-2.75)
2015-2016	136/1585	2.61 (2.19-3.07)	3.48 (2.92- 4.12)	727/2855	9.62 (8.94-10.34)	2.44 (2.24-2.64)
2017-2018	109/1701	2.34 (1.93-2.80)	3.53 (2.89- 4.26)	721/2735	11.71 (10.88-12.59)	2.98 (2.73-3.24)
2019-2021	87/2338	3.01 (2.42-3.69)	6.64 (5.30- 8.23)	540/3753	13.35 (12.25-14.51)	3.72 (3.36-4.11)
**CKD stage 5-ND**						
2011-2012	56/839	3.15 (2.40-4.05)	3.62 (2.75-4.65)	414/2334	10.79 (9.78-11.86)	2.88 (2.61-3.18)
2013-2014	52/659	3.96 (2.98-5.13)	4.90 (3.68-6.37)	328/1702	12.25 (10.97-13.63)	3.37 (3.00-3.77)
2015-2016	61/697	4.32 (3.33-5.50)	5.77 (4.43-7.36)	306/1681	12.25 (10.93-13.68)	3.10 (2.75-3.49)
2017-2018	50/771	3.65 (2.73-4.76)	5.51 (4.11-7.22)	321/1676	13.40 (11.99-14.92)	3.41 (3.02-3.83)
2019-2021	42/1090	4.02 (2.92-5.36)	8.88 (6.42-11.94)	308/2519	15.15 (13.52-16.90)	4.22 (3.72-4.77)
**Hemodialysis**						
2011-2012	201/1531	4.11 (3.56-4.70)	4.71 (4.08-5.40)	379/2274	5.46 (4.93-6.03)	1.46 (1.31-1.62)
2013-2014	32/326	3.04 (2.10-4.21)	3.76 (2.60-5.23)	182/1138	5.73 (4.94-6.61)	1.58 (1.35-1.82)
2015-2016	34/330	3.87 (2.71-5.32)	5.17 (3.62-7.13)	181/1271	5.34 (4.60-6.16)	1.35 (1.16-1.57)
2017-2018	36/312	5.32 (3.77-7.26)	8.04 (5.68-11.01)	158/1244	5.77 (4.92-6.72)	1.47 (1.24-1.72)
2019-2021	18/352	5.00 (3.03-7.68)	11.04 (6.67-17.05)	128/1857	6.63 (5.55-7.84)	1.85 (1.53-2.20)
**Peritoneal dialysis**						
2011-2012	18/228	4.87 (2.95-7.48)	5.59 (3.39-8.59)	88/921	5.31 (4.28-6.50)	1.42 (1.14-1.74)
2013-2014	5/98	2.60 (0.93-5.59)	3.23 (1.16-6.94)	60/621	5.77 (4.43-7.36)	1.59 (1.22-2.03)
2015-2016	8/91	5.07 (2.32-9.44)	6.76 (3.09-12.60)	60/655	5.44 (4.18-6.93)	1.38 (1.05-1.76)
2017-2018	5/95	4.16 (1.49-8.94)	6.29 (2.25-13.55)	58/632	6.55 (5.01-8.39)	1.67 (1.27-2.14)
2019-2021	2/134	2.01 (0.33-6.19)	4.42 (0.73-13.66)	45/1036	5.70 (4.19-7.52)	1.59 (1.16-2.11)

Standardized incidence rate ratio compared to a 1:10 age- and sex-matched general population. Standardized incidence rate ratios were calculated according to indirect standardization by taking the observed number of events divided by the expected number of events for CKD populations based on the observed rates in the age-, sex- matched general population, with 95% confidence intervals computed assuming a Poisson distribution.
CI: confidence intervals ; IR: incidence rate ; Std IRR: standardized incidence rate ratio.

Figure S1. Study design

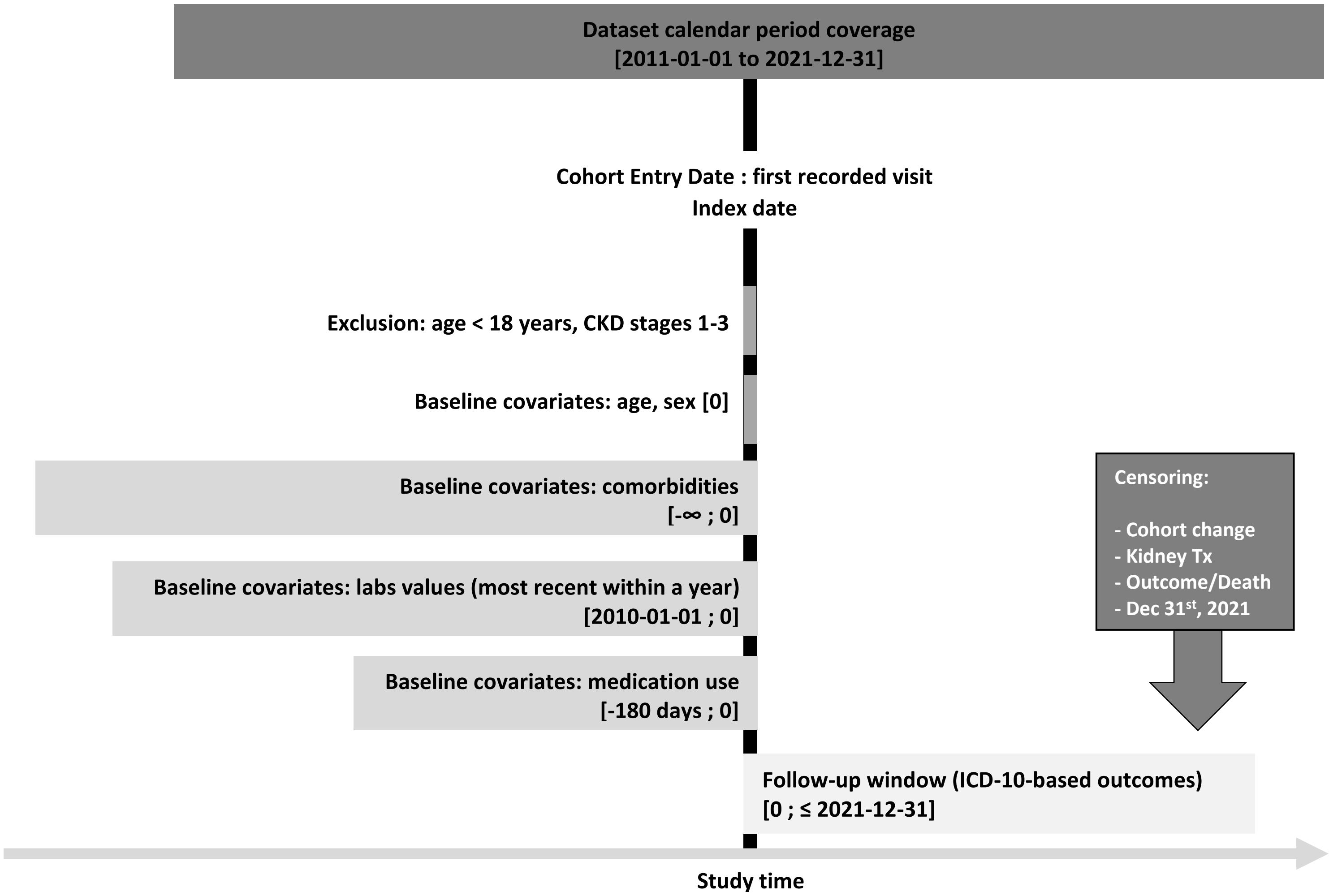

This graphical depiction of study design shows the different time windows used to define exposure, exclusion criteria, baseline covariates and outcomes. Adapted from *Schneeweiss et al, Ann Int Med, 2019*.

Figure S2. Flowchart for the study population

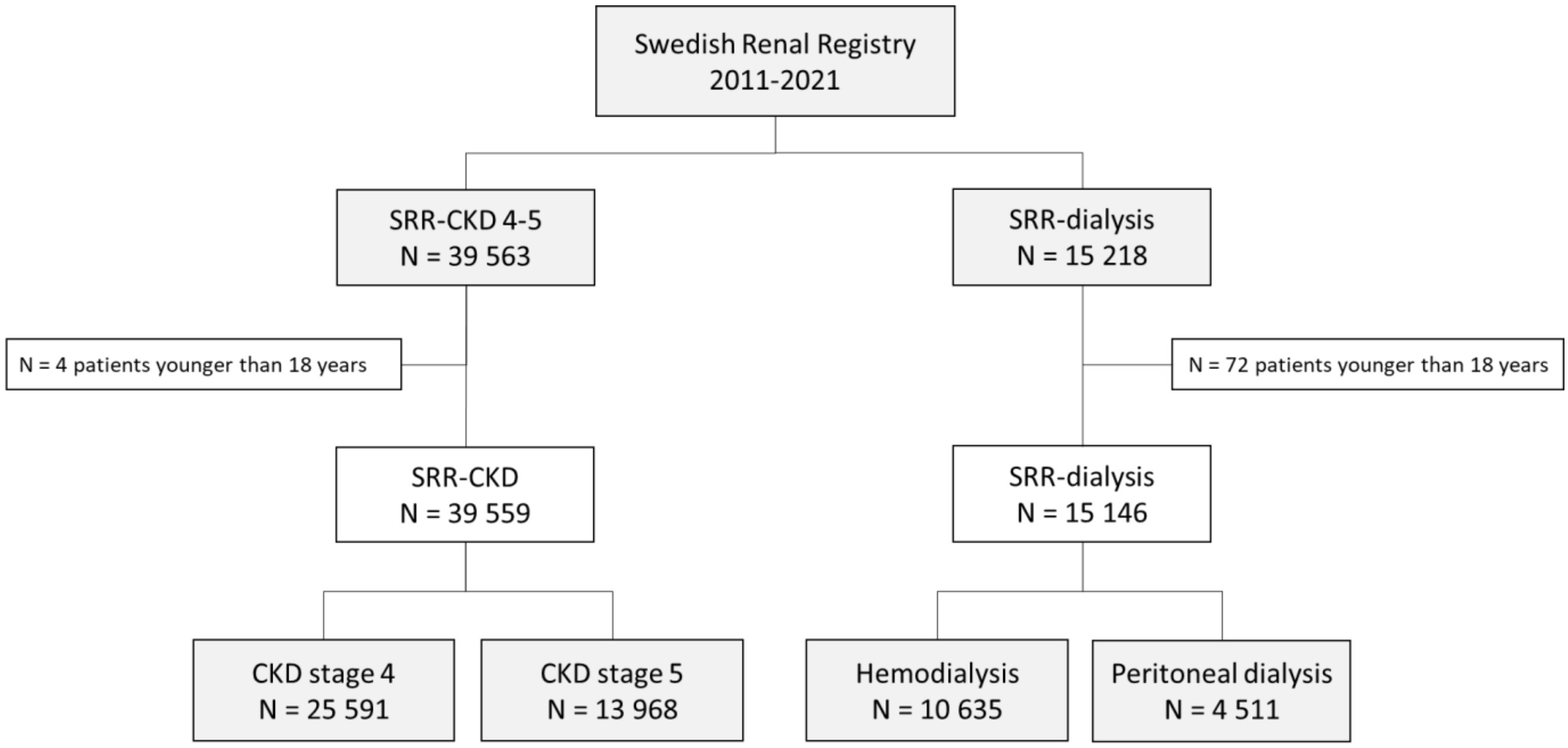

Figure S3. Underlying cause of heart failure-related death, by CKD cohort, in patients with HF as the main cause of death

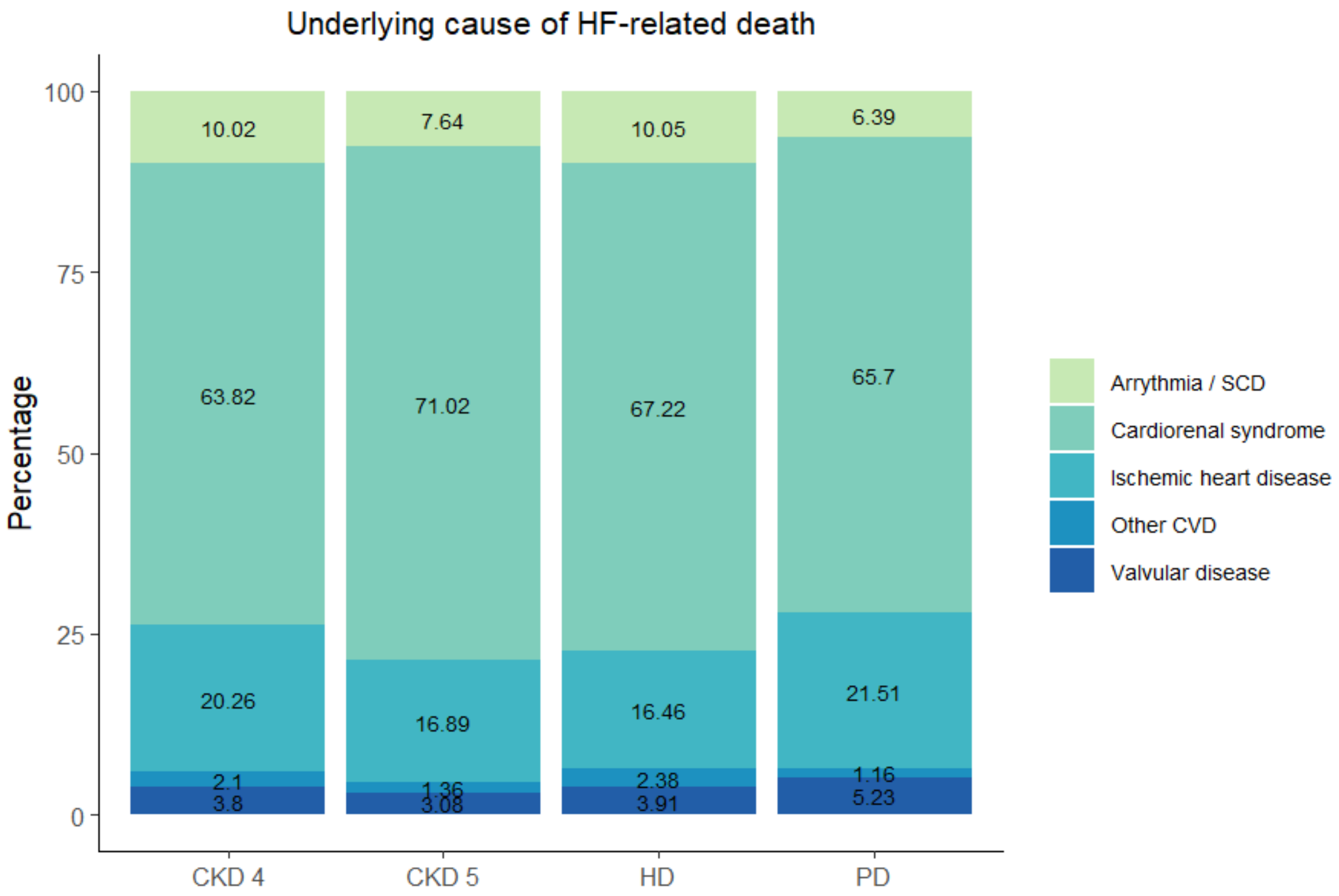

Figure S4. Prevalence of heart failure medications by sex and calendar year, in non-dialysis CKD.

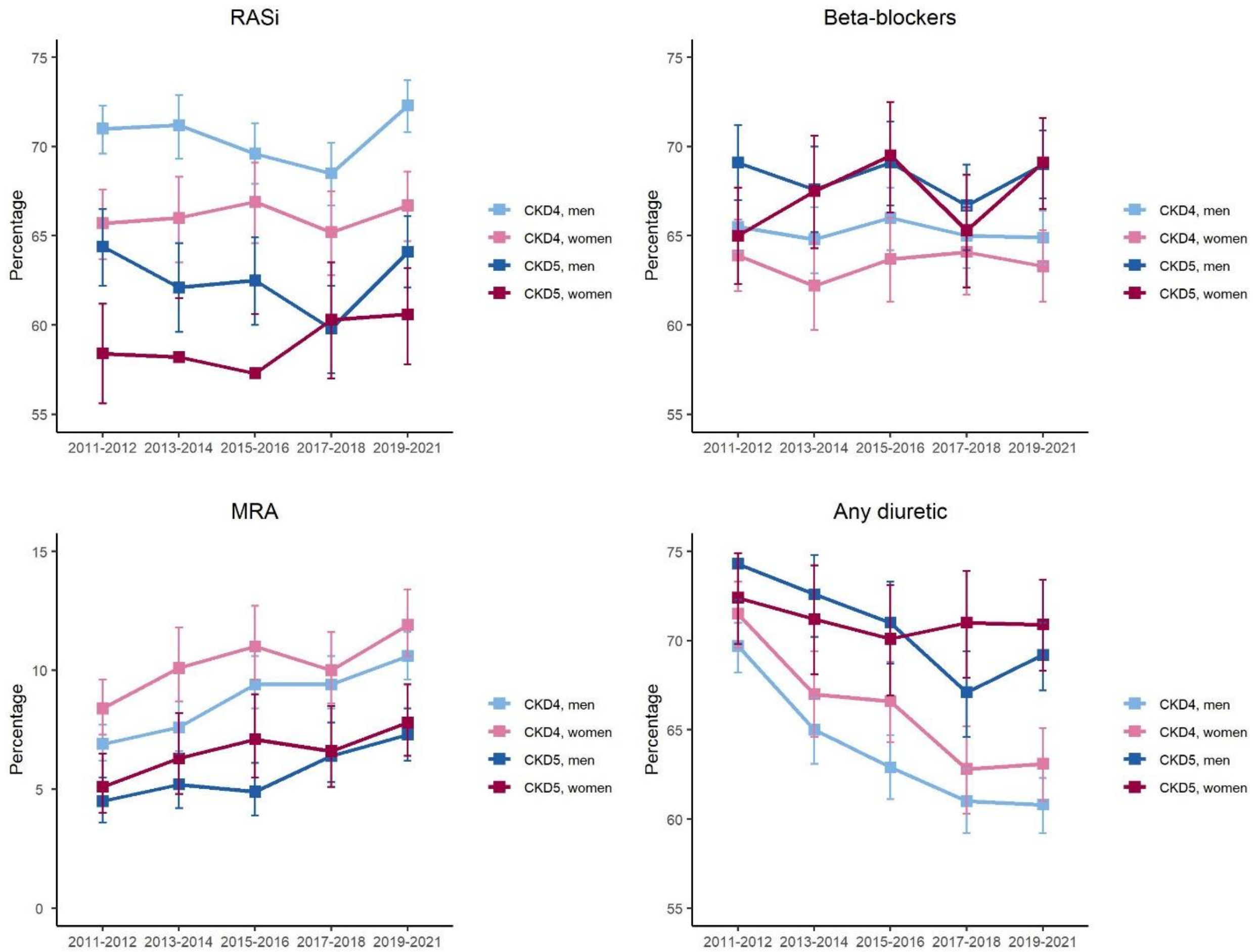

Error bars indicate 95% confidence intervals. CKD: chronic kidney disease ; HD: hemodialysis; MRA: mineralocorticoid receptor antagonist ; PD: peritoneal dialysis; RASi: renin-angiotensin system inhibitor.

Figure S5. Prevalence of heart failure medications by sex and calendar year, in dialysis CKD.

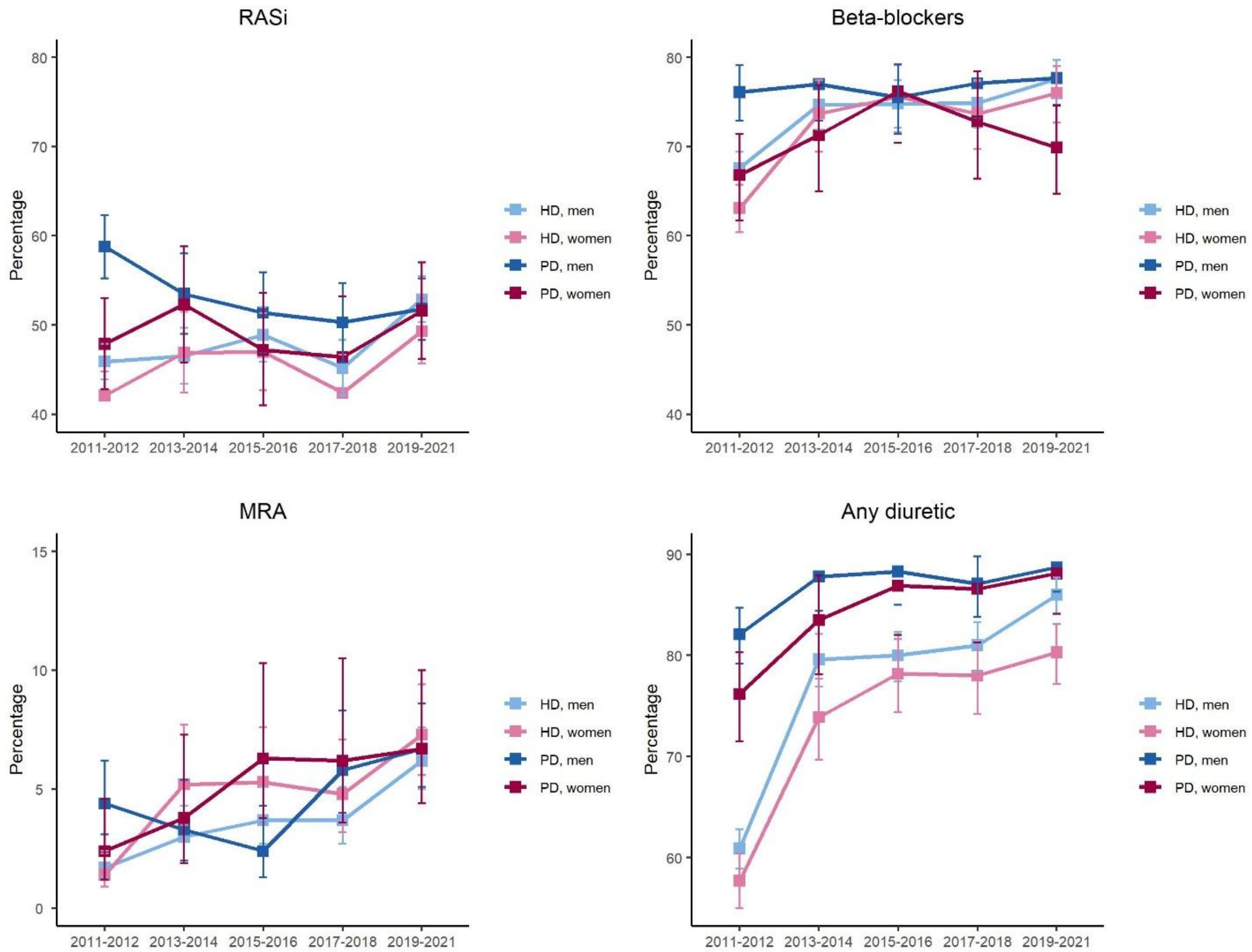

Error bars indicate 95% confidence intervals. CKD: chronic kidney disease ; HD: hemodialysis; MRA: mineralocorticoid receptor antagonist ; PD: peritoneal dialysis; RASi: renin-angiotensin system inhibitor.

STROBE Statement
checklist of items that should be included in reports of observational studies

	Item No.	Recommendation	Page No.	Relevant text from manuscript
**Title and abstract**	1	(*a*) Indicate the study's design with a commonly used term in the title or the abstract	1	
		(*b*) Provide in the abstract an informative and balanced summary of what was done and what was found	4	
**Introduction**				
Background/rationale	2	Explain the scientific background and rationale for the investigation being reported	5	
Objectives	3	State specific objectives, including any prespecified hypotheses	5	
**Methods**				
Study design	4	Present key elements of study design early in the paper	6, Fig S1	
Setting	5	Describe the setting, locations, and relevant dates, including periods of recruitment, exposure, follow-up, and data collection	6,7	
Participants	6	(*a*) *Cohort study*—Give the eligibility criteria, and the sources and methods of selection of participants. Describe methods of follow-up	6	
		Case-control study—Give the eligibility criteria, and the sources and methods of case ascertainment and control selection. Give the rationale for the choice of cases and controls	-	
		Cross-sectional study—Give the eligibility criteria, and the sources and methods of selection of participants	-	
		(*b*) *Cohort study*—For matched studies, give matching criteria and number of exposed and unexposed	6	Age-sex-matched general population for standardization
		Case-control study—For matched studies, give matching criteria and the number of controls per case	-	
Variables	7	Clearly define all outcomes, exposures, predictors, potential confounders, and effect modifiers. Give diagnostic criteria, if applicable	6,7	
Data sources/ measurement	8*	For each variable of interest, give sources of data and details of methods of assessment (measurement). Describe comparability of assessment methods if there is more than one group	6,7	
Bias	9	Describe any efforts to address potential sources of bias	-	
Study size	10	Explain how the study size was arrived at	6, Fig S2	

Continued on next page

Quantitative variables	11	Explain how quantitative variables were handled in the analyses. If applicable, describe which groupings were chosen and why	7	
Statistical methods	12	(*a*) Describe all statistical methods, including those used to control for confounding	7,8	
		(*b*) Describe any methods used to examine subgroups and interactions	7,8	
		(*c*) Explain how missing data were addressed	8	
		(*d*) *Cohort study*—If applicable, explain how loss to follow-up was addressed<br>*Case-control study*—If applicable, explain how matching of cases and controls was addressed<br>*Cross-sectional study*—If applicable, describe analytical methods taking account of sampling strategy	-	No loss to follow-up
		(*e*) Describe any sensitivity analyses	8	
**Results**				
Participants	13*	(a) Report numbers of individuals at each stage of study—eg numbers potentially eligible, examined for eligibility, confirmed eligible, included in the study, completing follow-up, and analysed	9, Fig S2	
		(b) Give reasons for non-participation at each stage	Fig S2	
		(c) Consider use of a flow diagram	Fig S2	
Descriptive data	14*	(a) Give characteristics of study participants (eg demographic, clinical, social) and information on exposures and potential confounders	9, Tables 1, S2-S5	
		(b) Indicate number of participants with missing data for each variable of interest	-	No missing data
		(c) *Cohort study*—Summarise follow-up time (eg, average and total amount)	Table 2	
Outcome data	15*	*Cohort study*—Report numbers of outcome events or summary measures over time	9,10, Table 2	
		Case-control study—Report numbers in each exposure category, or summary measures of exposure	-	

		Cross-sectional study—Report numbers of outcome events or summary measures	-
Main results	16	(*a*) Give unadjusted estimates and, if applicable, confounder-adjusted estimates and their precision (eg, 95% confidence interval). Make clear which confounders were adjusted for and why they were included	9,10
		(*b*) Report category boundaries when continuous variables were categorized	-
		(*c*) If relevant, consider translating estimates of relative risk into absolute risk for a meaningful time period	-
Other analyses	17	Report other analyses done—eg analyses of subgroups and interactions, and sensitivity analyses	10
**Discussion**			
Key results	18	Summarise key results with reference to study objectives	11
Limitations	19	Discuss limitations of the study, taking into account sources of potential bias or imprecision. Discuss both direction and magnitude of any potential bias	12
Interpretation	20	Give a cautious overall interpretation of results considering objectives, limitations, multiplicity of analyses, results from similar studies, and other relevant evidence	11-13
Generalisability	21	Discuss the generalisability (external validity) of the study results	13
**Other information**			
Funding	22	Give the source of funding and the role of the funders for the present study and, if applicable, for the original study on which the present article is based	14

*Give information separately for cases and controls in case-control studies and, if applicable, for exposed and unexposed groups in cohort and cross-sectional studies.

Note: An Explanation and Elaboration article discusses each checklist item and gives methodological background and published examples of transparent reporting. The STROBE checklist is best used in conjunction with this article (freely available on the Web sites of PLoS Medicine at http://www.plosmedicine.org/, Annals of Internal Medicine at http://www.annals.org/, and Epidemiology at http://www.epidem.com/). Information on the STROBE Initiative is available at www.strobe-statement.org.
